# CD8^+^ T cell differentiation status correlates with the feasibility of sustained unresponsiveness following oral immunotherapy

**DOI:** 10.1038/s41467-022-34222-8

**Published:** 2022-11-04

**Authors:** Abhinav Kaushik, Diane Dunham, Xiaorui Han, Evan Do, Sandra Andorf, Sheena Gupta, Andrea Fernandes, Laurie Elizabeth Kost, Sayantani B. Sindher, Wong Yu, Mindy Tsai, Robert Tibshirani, Scott D. Boyd, Manisha Desai, Holden T. Maecker, Stephen J. Galli, R. Sharon Chinthrajah, Rosemarie H. DeKruyff, Monali Manohar, Kari C. Nadeau

**Affiliations:** 1 Sean N. Parker Center for Allergy and Asthma Research, Department of Medicine, Stanford University School of Medicine, Stanford, CA USA; 2 Department of Pediatrics, University of Cincinnati College of Medicine, Cincinnati, OH USA; 3 Divisions of Biomedical Informatics and Allergy & Immunology, Cincinnati Children’s Hospital Medical Center, Cincinnati, OH USA; 4 Human Immune Monitoring Center, Institute for Immunity, Transplantation, and Infection, Stanford University School of Medicine, Stanford, CA USA; 5 Division of Allergy, Immunology, and Blood and Marrow Transplantation, Department of Pediatrics, University of California, San Francisco, CA USA; 6 Department of Pathology, Stanford University School of Medicine, Stanford, CA USA; 7 Department of Biomedical Data Science, Stanford University, Stanford, CA USA; 8 Department of Microbiology and Immunology, Stanford University School of Medicine, Stanford, CA USA

**Keywords:** CD8-positive T cells, Immunotherapy, Lymphocyte differentiation, T-helper 2 cells

## Abstract

While food allergy oral immunotherapy (OIT) can provide safe and effective desensitization (DS), the immune mechanisms underlying development of sustained unresponsiveness (SU) following a period of avoidance are largely unknown. Here, we compare high dimensional phenotypes of innate and adaptive immune cell subsets of participants in a previously reported, phase 2 randomized, controlled, peanut OIT trial who achieved SU vs. DS (no vs. with allergic reactions upon food challenge after a withdrawal period; *n* = 21 vs. 30 respectively among total 120 intent-to-treat participants). Lower frequencies of naïve CD8^+^ T cells and terminally differentiated CD57^+^CD8^+^ T cell subsets at baseline (pre-OIT) are associated with SU. Frequency of naïve CD8^+^ T cells shows a significant positive correlation with peanut-specific and Ara h 2-specific IgE levels at baseline. Higher frequencies of IL-4^+^ and IFNγ^+^ CD4^+^ T cells post-OIT are negatively correlated with SU. Our findings provide evidence that an immune signature consisting of certain CD8^+^ T cell subset frequencies is potentially predictive of SU following OIT.

## Abstract (web summary)

Oral immunotherapy (OIT) clinical trials have helped a subset of participants achieve sustained unresponsiveness (SU) to the cognate allergen. Here the authors analyse immune cells from participants from one peanut OIT trial and show that CD8^+^ T cell differentiation status at baseline may help to predict the likelihood of achieving SU.

## Introduction

Oral immunotherapy (OIT), during which food allergens are gradually introduced at increasing doses until a maintenance dose is reached, has been successful in desensitizing patients to offending food antigens and many trials administering peanut OIT have been performed^1–3^. While these trials have demonstrated the efficacy of peanut OIT to desensitize participants to peanut, the proportion of such desensitized participants achieving sustained unresponsiveness (SU), i.e., a lack of clinical reactivity following a period of OIT discontinuation is highly variable^1–3^. Moreover, while some aspects of the immune mechanisms resulting in desensitization (i.e., DS, for the purposes of this study, defined by the recurrence of any allergic reaction upon food challenge after a period of withdrawal post-successful OIT outcome) have been largely elucidated, the immune pathways or biomarkers that may distinguish SU vs. DS are yet to be fully characterized^4–7^.

To address this issue and to gain insight into mechanisms underlying development of SU, we performed a comprehensive post-hoc mechanistic analysis of peripheral blood mononuclear cells (PBMCs) and plasma from participants in our phase 2 OIT POISED study (Peanut Oral Immunotherapy: Safety, Efficacy, Discovery) (NCT 02103270). The design, objectives, and primary outcomes of this randomized, double-blind, placebo-controlled (DBPC) clinical trial have been previously published^8^. Briefly, in this study, after dosage build-up over ~52 weeks, peanut-allergic patients were maintained on 4000 mg peanut protein daily for another 52 weeks, while a blinded placebo group received oat flour. At week 104, 80 (98.77%) of per-protocol active participants passed the DBPC food challenge (DBPCFC) and peanut ingestion was withdrawn in a group of 51 blinded participants (i.e., peanut avoidance group) for 12 weeks. At week 117, 21 (41.2%) of per-protocol participants in the peanut avoidance group passed the 4000 mg DBPCFC without any allergic reaction, thus demonstrating SU. Those 21 participants continued to have DBPCFCs every 3 months and were allowed to continue peanut OIT discontinuation if they passed a DBPCFC. At week 156, 12 months after peanut discontinuation, only 8 participants passed the DBPCFCs (i.e., had achieved long-term SU with no allergic reaction upon food challenge).

We recently reported higher frequencies of peanut-reactive (pr) CD8^+^ T cells in peanut-allergic individuals and demonstrated interactions between pr CD8^+^ and Th2 cells leading to enhancement of allergic pathways^9,10^. Based on these findings, we postulated a pr CD8^+^ T cell-based mechanism in distinguishing SU vs. DS outcomes. Furthermore, our group and others have demonstrated distinct functions of innate immune cells in food allergy^11,12^, and so we further hypothesized that pr CD8^+^ T cells along with innate immune cell subsets likely impact the possibility achieving SU.

To test these hypotheses, we perform high dimensional functional immunophenotyping of peanut-stimulated, PMA/Ionomycin-stimulated, and unstimulated PBMCs with mass cytometry, and plasma and peanut-stimulated PBMC supernatants with Luminex technology from all per-protocol trial participants. We quantitate immune changes from SU (i.e., SU defined as those who had no allergic reaction at the DBPCFC at week 117 or later) vs. DS (i.e., those who had allergic reactions to the DBPCFC at week 117) at week 117. To identify potential biomarkers predictive of SU, we examine differences in immune profiles of SU vs. DS participants at baseline (BL, i.e., pre-OIT) as well as at the end of OIT at week 104 (i.e., after successful desensitization). One of the primary baseline inclusion criterion for study participation was tolerance to ≤500 mg of cumulative peanut protein dose during the DBPCFC at the screening stage. The participants who successfully passed 4000 mg peanut OFC at week 104, but failed at a peanut dose of ≤500 mg at week 117 represented a subset having relapsed to the baseline threshold following mere 12 weeks of avoidance. Thus, as a nested analysis of participants at the ends of SU vs. DS outcome spectrum, we compare a subset of DS participants, who failed week 117 DBPCFC at a peanut protein dose ≤500 mg (*n* = 7) with the 8 SU participants, who passed all DBPCFCs up to week 156. We also examine peanut OIT-induced immune changes by comparing active vs. placebo groups at week 104.

Our results show that lower frequencies of naive CD8^+^ T cells and terminally differentiated, CD57^+^ pr CD8^+^ T cells at BL are strongly associated with SU. On the other hand, higher frequencies of IL-4^+^ and IFNγ^+^ CD4^+^ T cells post-OIT are negatively correlated with SU. In summary, we discover a distinct CD8^+^ T cell pattern in vivo that is associated with the impairment of achieving SU during food allergy OIT. In addition, our data confirm the attenuation of Th2 phenotype post-OIT.

## Results

### Peanut OIT leads to attenuation of Type 2 phenotype

**Fig. 1:**
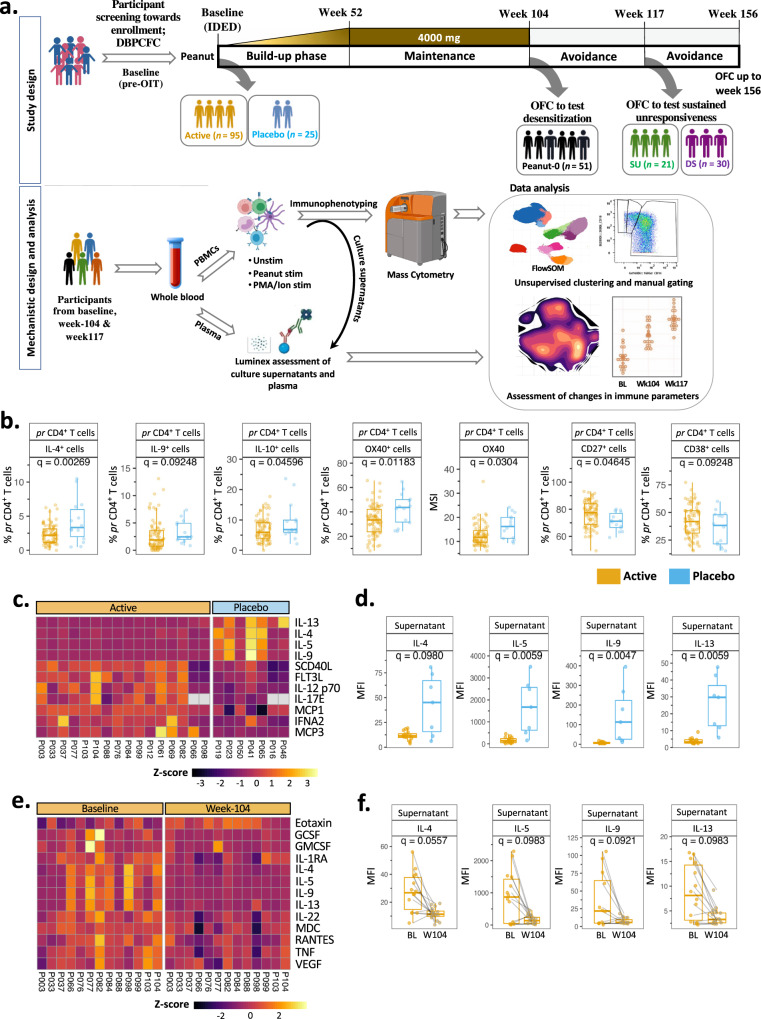
Peanut OIT effects an immune shift away from Th2 phenotype. **a** Top panel: schematic representation of POISED study timeline and sample collection time points. Bottom panel: depiction of approach for mechanistic analyses. Here, “SU” stands for sustained unresponsive and “DS” stands for desensitized alone participants. Illustration created with BioRender.com. **b** Immune cell subsets among peanut-stimulated week 104 PBMCs from participants on active peanut OIT (*n* = 80) were compared against those treated with placebo (*n* = 15). Significant frequency (i.e., percentage) and expression (i.e., MSI) changes among peanut-reactive (pr) CD4^+^ T cells are shown. **c**, **d** Significant changes in peanut-stimulated week 104 PBMC culture supernatant of active participants (*n* = 16) compared to those treated with placebo (*n* = 7) as a heatmap (**c**) and as individual boxplots (**d**). **e**, **f** Significant changes in peanut-stimulated week 104 PBMC culture supernatant of active participants (*n* = 13) compared to BL as a heatmap (**e**) and individual boxplots (**f**). In the boxplots, medians are shown, and the “hinges” represent the first and third quartile. The whiskers are the smallest and largest values after exclusion of outliers (greater than the 75th percentile plus 1.5 times the interquartile range (IQR), or less than 25th percentile minus 1.5 times the IQR). *q* values were computed using the *χ*^2^ tests in mixed-effects models, adjusted for multiple hypothesis testing using the Benjamini and Hochberg method. Source data are provided as a Source Data file.

Figure 1a depicts the POISED study design (top panel) and the workflow for mechanistic assays (bottom panel). High dimensional intracellular and extracellular proteins in peanut-stimulated PBMCs from participants on active peanut OIT vs. placebo were quantified by mass cytometry at week 104.

The frequencies of IL-4^+^, IL-9^+^ and IL-10^+^ peanut-reactive (pr) CD4^+^ T cells (i.e., CD69^+^CD40L^+^CD4^+^ T cells) among total CD4^+^ T cells were significantly reduced in participants on active OIT (Fig. 1b; *q* = 0.002, 0.09 and 0.04, respectively). Peanut-reactive CD4^+^ cells also demonstrated a reduced frequency and expression of Th2-polarization surrogate marker OX40 in active vs. placebo groups (Fig. 1b; *q* = 0.01 and 0.03, respectively), indicating downregulation of the Type 2 phenotype in the active peanut OIT-treated, desensitized participants. Peanut-reactive CD4^+^ T cells also demonstrated increased frequencies of subpopulations expressing the costimulatory molecule CD27 and the activation marker CD38 in active vs. placebo (Fig. 1b; *q* = 0.046 and 0.092, respectively). Similar differences in OX40 and CD27 expression were observed in active vs. placebo comparison of pr CD4^+^ cell clusters identified through FlowSOM-based unsupervised clustering (Supplementary Fig. 1a, b; *q* = 0.01 and 0.006, respectively). Expression of other functional markers evaluated in pr CD4^+^ T cells including IL-17, LAP (TGF-β) and IFN-γ and frequencies of pr CD4^+^ and pr CD8^+^ T cells among peanut-stimulated PBMCs were similar in active vs. placebo comparison at week 104 (Supplementary Fig. 1c; *q* > 0.1). Also, frequencies of all the manually gated immune subpopulations examined within the scope of our mass cytometry panel including Treg (CD25^hi^CD127^-^CD4^+^), NKT (CD56^+^CD3^+^αβ T), naive γδ (CD45RA^+^CCR7^+^γδTCR^+^ CD3^+^) T cells, and plasmablasts (CD27^hi^CD38^hi^HLA-DR^+^CD19^+^CD3^-^) among peanut-stimulated and unstimulated PBMCs were comparable across active and placebo participants at week 104 (Supplementary Fig. 1d; *q* > 0.1).

Analysis of supernatants of peanut-stimulated PBMCs by Luminex assay showed that expression of the Type 2 cytokines IL-4, IL-5, IL-9 and IL-13 was significantly decreased at week 104 in active vs. placebo (*q* = 0.09, 0.006, 0.005 and 0.006, respectively) (Fig. 1c, d). This marked decrease in IL-4, IL-5, IL-9 and IL-13 was also evident when comparing their levels in peanut-stimulated culture supernatants of active participants’ PBMCs at week 104 with those at BL (*q* = 0.05, 0.09, 0.09 and 0.09, respectively) (Fig. 1e, f), further supporting a shift away from a Type 2 profile. Apart from Type 2 cytokine changes, peanut-stimulated PBMC culture supernatant from active participants showed an increase in Monocyte Chemoattractant Proteins MCP1 and MCP3 (active vs. placebo, *q* = 0.02 and 0.01, respectively), as well as IL-12 p70 and FLT3 Ligand (*q* = 0.03 and 0.07, respectively) compared with placebo (Supplementary Fig. 1e). In contrast, peanut-stimulated PBMC culture supernatants from active participants showed a marked increase in the eosinophil chemotactic protein Eotaxin at week 104 vs. BL (*q* = 0.05), and a concomitant decrease in proinflammatory cytokines including RANTES, TNF, and IL-22 (Supplementary Fig. 1f; *q* = 0.098, 0.065 and 0.059, respectively).

### CD8^+^ T cell subset distinctions as a hallmark of SU vs. DS outcome

**Fig. 2:**
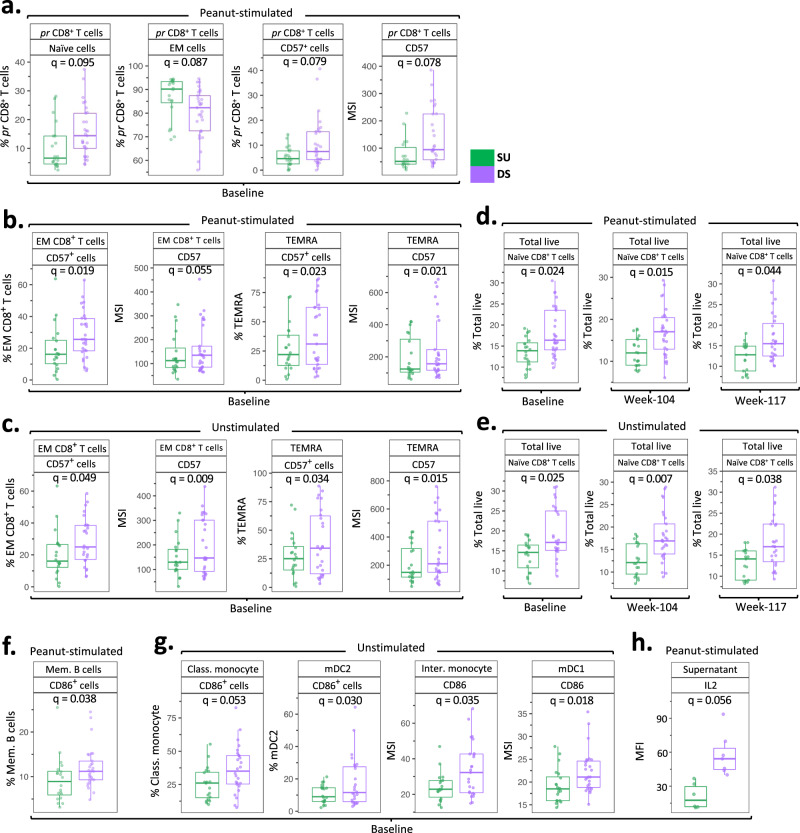
CD8^+^ T cell differentiation status distinguishes SU participants from DS. Immune cell subsets among peanut-stimulated (**a**, **b**, **d**, **f**) and unstimulated (**c**, **e**, **g**) PBMCs from SU participants were compared with DS participants at BL, week 104, and week 117. **a**–**c** Significant frequency and CD57 expression differences among pr CD8^+^ T cells (**a**), and peanut non-reactive CD8^+^ T cells (**b**) among peanut-stimulated (SU, *n* = 20; DS, *n* = 29), and unstimulated (**c**) (SU, *n* = 20; DS, *n* = 26), BL PBMCs. **d**, **e** Significant differences in the naive CD8^+^ T cell frequency in peanut-stimulated (**d**) and unstimulated (**e**) PBMCs at BL (pea stim: SU, *n* = 20, DS, *n* = 29; unstim: SU, *n* = 20, DS, *n* = 26), week 104 (pea stim: SU, *n* = 21, DS, *n* = 30; unstim: SU, *n* = 20, DS, *n* = 29), and week 117 (pea stim: SU, *n* = 17, DS, *n* = 27; unstim: SU, *n* = 17, DS, *n* = 27). **f**, **g** Significant differences in the frequency or expression of CD86 in memory B cells among peanut-stimulated PBMCs (**f**) and APC subsets among unstimulated PBMCs (**g**) at BL (pea stim: SU, *n* = 20, DS, *n* = 29; unstim: SU, *n* = 20, DS, *n* = 26). **h** IL-2 levels in peanut-stimulated BL PBMC culture supernatants from SU (*n* = 7) vs. DS (*n* = 7) participants. Boxplots show medians and “hinges” represent the first and third quartile. Whiskers denote the smallest and largest values after outlier exclusion. *q* values computed using the *χ*^2^ tests in mixed-effects models, adjusted for multiple hypothesis testing using the Benjamini and Hochberg method. Source data are provided as a Source Data file.

While all 51 per-protocol active participants randomized into the avoidance group were successfully desensitized at week 104, 12 weeks of peanut avoidance thereafter led to two distinct clinical outcomes, viz., SU (*n* = 21) vs. DS (*n* = 30) (no vs. with allergic reactions on the 4000 mg DBPCFC at week 117, respectively). To identify changes in immune markers or cell type frequencies that might identify SU participants, we compared immune changes in peanut-stimulated and unstimulated PBMCs from SU participants with those from DS at BL, week 104, and week 117. Interestingly, in both peanut-stimulated and unstimulated PBMCs, differences distinguishing SU vs. DS were identified within CD8^+^ T cell subsets but not in CD4^+^ T cells. Peanut-reactive (pr) CD8^+^ T cells (i.e., CD69^+^CD8^+^) among peanut-stimulated PBMCs of SU participants were primarily of effector memory (EM; CD45RA^-^CCR7^-^) phenotype while those from DS participants exhibited a naive (CD45RA^+^CCR7^+^) phenotype (Fig. 2a). Also, pr CD8^+^ T cells from DS participants had a significantly higher frequency and expression of CD57 (*q* = 0.079 and 0.079, respectively), a marker of terminal differentiation (Fig. 2a). Similarly, EM and TEMRA (CD45RA^+^CCR7) CD8^+^ T cell subsets of peanut non-reactive (i.e., CD69^-^) CD8^+^ T cells among peanut-stimulated PBMCs from DS participants showed a significantly higher frequency and expression of CD57 (Fig. 2b; *q* = 0.019, 0.05, 0.02 and 0.02, respectively). This CD57 expression pattern among EM and TEMRA CD8^+^ cells was evident in unstimulated PBMCs as well (Fig. 2c; SU vs. DS *q* = 0.05, 0.009, 0.03 and 0.01, respectively). Interestingly, CD56^dim^CD16^+^ NK cells, a subset involved in host protective immunity through cytolysis along with CD8^+^ T cells, from DS participants also showed an elevated frequency and expression of CD57 at BL and at week 117 in peanut-stimulated (Supplementary Fig. 2a; *q* = 0.059 and 0.068, respectively) and unstimulated PBMCs (Supplementary Fig. 2a; *q* = 0.02 and 0.01, respectively).

In addition, among peanut non-reactive CD8^+^ T cells in peanut-stimulated PBMCs, the frequency of naive CD8^+^ cells was substantially lower in SU vs. DS participants at BL, week 104 and week 117 (Fig. 2d; *q* = 0.02, 0.01 and 0.04, respectively). Unstimulated SU vs. DS BL, week 104 and week 117 PBMCs also reflected these differences in naive CD8^+^ T cell frequency (Fig. 2e; *q* = 0.02, 0.008 and 0.03, respectively). A significantly reduced frequency of CD8^+^ naive T cells among total live cells was also detected in SU vs. DS participants at BL, week 104 and week 117 using unsupervised clustering of peanut-stimulated PBMCs (*q* = 0.09, 0.009 and 0.09, respectively), and unstimulated PBMCs (*q* = 0.09, 0.01 and 0.06, respectively) (Supplementary Fig. 2b–d). On the contrary, SU participants showed a markedly higher frequency of peanut non-reactive EM CD8^+^ cells among peanut-stimulated and ustimulated total CD8^+^ cells at week 104, and week 117 PBMCs compared to DS participants (Supplementary Fig. 2e; *q* = 0.02, 0.03 respectively, and Supplementary Fig. 2f*q* = 0.025 each). This trend of higher EM CD8^+^ T cell frequency could be observed at BL as well.

Frequencies of pr CD4^+^ and pr CD8^+^ T cells among peanut-stimulated PBMCs and expression of the evaluated functional markers within these subsets were similar between SU and DS participants at BL, week 104, and week 117 (Supplementary Fig. 3a–h; *q* > 0.1). Particularly, while there were no significant differences in the respective frequencies and no significant differences in the expression of IL-4, IL-9, and IL-10 between SU vs. DS participants at week 117, SU participants had lower frequencies of IL-4^+^ and IL-9^+^ pr CD4^+^ T cells and lower expression of IL-4 compared to DS participants at BL, week 104, as well as week 117 (Supplementary Fig. 3b–d). The frequencies of all the manually gated immune subpopulations examined within the scope of our mass cytometry panel including Treg, NKT, naive γδ T cells and plasmablasts among peanut-stimulated and unstimulated PBMCs were comparable across SU and DS participants at BL, week 104 and week 117 (Supplementary Fig. 3i–l; *q* > 0.1).

Eligible participants further avoided peanut consumption past week 117 in a blinded protocol, and eight participants in this group successfully tolerated a 4000 mg peanut DBPCFC at week 156, thereby having achieved “long-term” SU (henceforth termed, SU week 156) following 1 year of peanut avoidance (Supplementary Fig. 4a). Immune changes in these SU week 156 participants were compared with those of participants who failed week 117 DBPCFC at a dose lower than 500 mg peanut (termed DS ≤ 500 mg; *n* = 7). Such subset selection afforded immune evaluation of participants representing each end of the treatment outcome spectrum. The frequency of IL-9^+^ pr CD4^+^ T cells at week 104 and expression of IL-9 among pr CD4^+^ T cells at week 117 were significantly lower in the SU week 156 vs. DS ≤ 500 mg (Supplementary Fig. 4b; *q* = 0.03 and 0.05, respectively). While the CD57 expression and frequency among CD8 subsets did not significantly differ in SU week 156 vs. DS ≤ 500 mg, peanut-stimulated and unstimulated week 117 PBMCs from SU week 156 exhibited a lower frequency of CD57^+^CD56^dim^CD16^+^ NK cells than DS ≤ 500 mg (Supplementary Fig. 4c; *q* = 0.05 and 0.06, respectively). The frequency of naive CD8^+^ T cells in total live peanut-stimulated and unstimulated PBMCs from SU week 156 participants was substantially lower at week 104 (Supplementary Fig. 4d, e; *q* = 0.002 and 0.003, respectively) and week 117 (Supplementary Fig. 4d, e; *q* = 0.002 and 0.006, respectively) compared with those in the DS ≤ 500 mg group. Notably, on comparing the frequencies of naive CD8^+^ T cells in SU week 156 (*n* = 8), DS week 130 to 156 (i.e., per-protocol participants, who passed at week 117, but failed at week 130/143/156; *n* = 10), DS week 117 > 500 mg (*n* = 23), and DS week 117 ≤ 500 mg (*n* = 7) at BL, week 104, and week 117, a trend of gradual increase in the frequency of naive CD8^+^ cells going from SU week 156 to DS week 117 ≤ 500 mg was observed (Supplementary Fig. 4f, g).

### Differences in CD86 expression on APC subsets from SU vs. DS participants

Besides CD8^+^ T cell subsets, we found significant differences in CD86 expression on APC subsets in SU vs. DS. The frequencies of CD86 expressing memory B (CD27^+^CD38^+^HLA-DR^+^CD19^+^CD3^-^) cells among peanut-stimulated PBMCs (Fig. 2f; *q* = 0.039) and that among classical monocytes (CD14^+^CD16^-^HLA-DR^+^) and myeloid dendritic cells 2 (mDC2; CD11c^+^CD123^-^CD14^-^HLA-DR^+^) among unstimulated PBMCs from SU were markedly lower than DS at BL (Fig. 2g; *q* = 0.05 and 0.03 respectively). Intermediate monocytes (CD14^+^CD16^+^HLA-DR^+^) and myeloid dendritic cells 1 (mDC1; CD11c^+^CD123^+^CD14^-^HLA-DR^+^) among unstimulated PBMCs from SU participants also showed a lower expression of CD86 (Fig. 2g; *q* = 0.03 and 0.01, respectively). In addition, a singular Luminex readout distinctive of SU vs. DS was lower levels of IL-2 in peanut-stimulated PBMC culture supernatants at BL (Fig. 2h; *q* = 0.056).

Similarly, reduced CD86^+^ frequency or CD86 expression was seen in APC subsets among PBMCs from SU week 156 group compared to the DS ≤ 500 mg group of participants. The frequency of CD86^+^ classical monocytes and memory B cells among peanut-stimulated week 104 PBMCs, and frequency of CD86^+^ classical monocytes, intermediate monocytes and mDC1s among unstimulated BL PBMCs, was significantly lower in SU week 156 vs. DS ≤ 500 mg (Supplementary Fig. 4h, i; *q* = 0.067, 0.080, and 0.007, 0.06 and 0.02, respectively). Classical monocytes, mDC1, and mDC2 subsets at BL from SU week 156 participants also showed lower expression of CD86 than DS ≤ 500 mg participants (Supplementary Fig. 4i; *q* = 0.03, 0.02 and 0.02, respectively).

### Correlations between distinctive immune subsets and specific immunoglobulin readouts

**Fig. 3:**
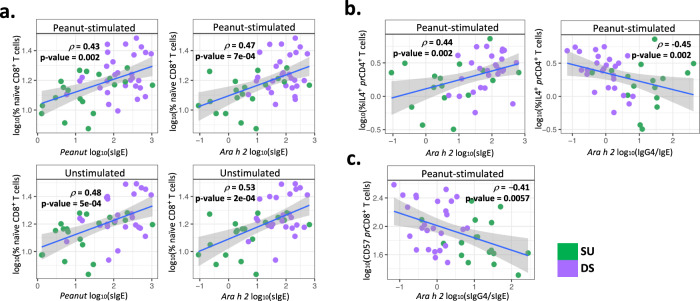
Naive CD8^+^, IL-4^+^ pr CD4^+^, and CD57^+^ pr CD8^+^ T cell subsets at baseline significantly correlate with peanut and Ara h 2-specific immunoglobulin readouts. **a** Spearman correlations of frequency of naive CD8^+^ T cells among total live cells from peanut-stimulated (top panels) and unstimulated (bottom panels) BL PBMCs with peanut-specific, and Ara h 2-specific IgE. **b** Spearman correlations of frequency of peanut-reactive IL-4^+^ CD4^+^ cells among total pr CD4^+^ T cells in peanut-stimulated PBMCs with Ara h 2-specific IgE and Ara h 2-specific IgG4/IgE ratio at BL. **c** Spearman correlation of frequency of peanut-reactive CD57^+^ CD8^+^ cells within total pr CD8^+^ T cells in peanut-stimulated PBMCs with Ara h 2-specific IgG4/IgE ratio at BL (pea stim: SU, *n* = 20, DS, *n* = 29; unstim: SU, *n* = 20, DS, *n* = 26). Data are presented as the mean ± SE. *p* values in the figure were determined by an unpaired two-tailed Student’s *t* test. Plots display the correlation coefficient (*ρ*) and linear regression line with a 95% confidence interval shading. Source data are provided as a Source Data file.

We and others have shown that an increased peanut-specific and peanut component-specific IgG4/IgE ratio driven by increased allergen-specific IgG4 is associated with induction of SU^8,13,14^. Analyses were thus performed to probe correlations of peanut- or peanut component-specific IgE, IgG4 and IgG4/IgE antibody levels at BL with immune cell subset frequencies, which we had observed to be associated with SU in this study. We observed a positive correlation of the frequency of naive CD8^+^ T cells in peanut-stimulated PBMCs with peanut-specific IgE (Fig. 3a; *p* = 0.43, *p* = 0.002) as well as Ara h 2-specific IgE (Fig. 3a; *p* = 0.47, *p* < 0.001) levels at BL. Such positive correlation with peanut- and Ara h 2-specific IgE was also observed with naive CD8^+^ T cells among unstimulated PBMCs (Fig. 3a; *p* = 0.48 and 0.53 respectively; *p* < 0.001 for both). Notably, participants who demonstrated SU at week 117 had both significantly lower levels of peanut-specific IgE at BL and significantly lower numbers of naive CD8^+^ T cells at BL (Fig. 2a) than participants that were DS (Fig. 3a). On the other hand, the BL frequency of EM CD8^+^ cells among peanut-stimulated total CD8^+^ cells exhibited a significant negative correlation with BL peanut-specific IgE (Supplementary Fig. 5a; *p* = −0.43, *p* = 0.0016). Similarly, a significant negative correlation was observed between the frequency of EM CD8^+^ cells among peanut-stimulated total CD8^+^ cells and Ara h 2-specific IgE at BL (Supplementary Fig. 5b; *p* = −0.46, *p* = 0.0008). To further dissect this intriguing link between relative frequencies of naive vs. EM CD8^+^ T cell subsets with serological readouts and hence SU as an outcome, we probed a convenience sample set of week 52 PBMCs from 5 SU vs. DS participants each. Interestingly, unstimulated and PMA/Ionomycin (PMAi)-stimulated CD8^+^ cells from SU participants exhibited a significantly higher frequency of the key cytolytic effector molecule, Granzyme B (GzB)-expressing subset compared to DS participants. A significantly higher GzB expression was observed in PMAi-stimulated CD8^+^ cells from SU participants as well compared to DS participants (Supplementary Fig. 5c; *p* < 0.01). Overall, EM CD8^+^ followed by TEMRA CD8^+^ T cells primarily contributed to GzB production. Nevertheless, DS participants had a lower mean proportion of GzB^+^ EM CD8^+^ cells (43.28% vs. 54.14%) and a higher mean proportion of GzB^+^ naive CD8^+^ cells (18.46 vs. 9.44%) compared to SU participants (Supplementary Fig. 5d).

As expected, we found that the frequency of IL-4^+^ peanut-reactive CD4^+^ T cells was positively correlated with the level of Ara h 2-specific IgE (Fig. 3b; *p* = 0.44, *p* = 0.002) and negatively correlated with the ratio of Ara h 2-specific IgG4/IgE at BL (Fig. 3b; *p* = −0.45, *p* = 0.002). Those participants who developed SU displayed lower values of Ara h 2-specific IgE and higher ratios of Ara h 2-specific IgG4/IgE^1^. Also, the frequency of CD57^+^ pr CD8^+^ T cells at BL negatively correlated with peanut and Ara h 2-specific IgG4/IgE levels (Fig. 3c; *p* = −0.41, *p* = 0.005).

### Supervised learning-based identification of features predictive of SU vs. DS outcome

As an independent approach to validate the above-mentioned observations, we applied supervised learning on the BL CyTOF readouts to identify features that could potentially predict the outcome as SU vs. DS (Supplementary Fig. 6a, b). We thus simulated the frequencies and marker expressions of manually gated immune cell subsets in all the participants studied (300 times data simulation). Each of the simulated dataset was then used to build an elastic net machine learning (ML) model, from which the most important features were elucidated (see Methods). Here, the data simulation not only enabled probing of the small sample size (*n* = 46 at BL), but also facilitated capturing the important features conserved in the respective group even after constricted random sampling^15^. The elastic net model could correctly predict the SU participants with an average AUC of 0.94. In agreement with CyTOF analyses, the most informative features included frequencies of naive CD8^+^ T cells and CD57^+^ TEMRA CD8^+^ T cells thus endorsing the current findings.

### Lower frequencies of certain IFNγ^+^ T cell subsets post-OIT in SU participants

**Fig. 4:**
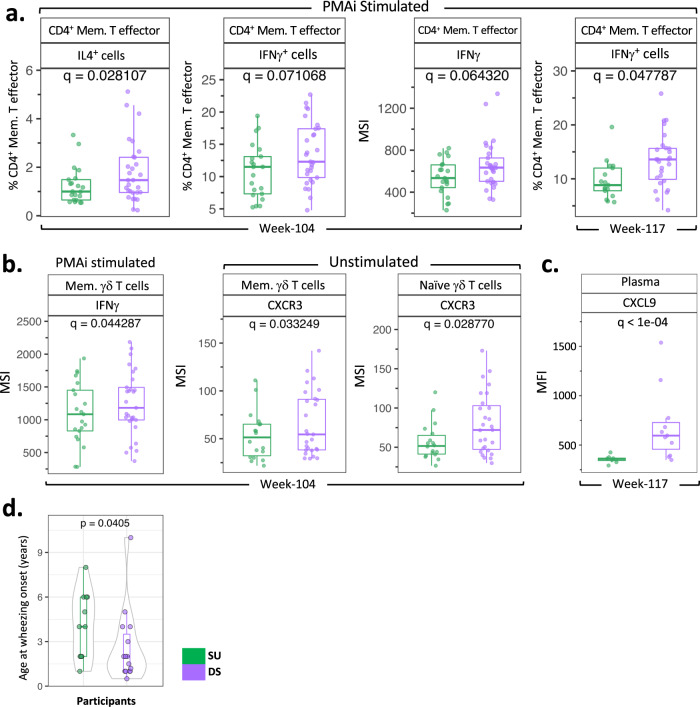
Higher frequency and expression of IFNγ as an additional distinctive feature of DS vs. SU participants. **a** Frequency of IL-4^+^ CD4^+^ memory T effectors at week 104, frequencies of IFNγ^+^ CD4^+^ memory T effectors at week 104 and week 117, and expression of IFNγ in CD4^+^ memory T effectors at week 104 in PMAi-stimulated PBMCs from SU vs. DS [week 104 (PMAi stim: SU, *n* = 21, DS, *n* = 29); week 117 (PMAi stim: SU, *n* = 17, DS, *n* = 27)]. **b** Expression of IFNγ in memory γδ T cells among PMAi-stimulated week 104 PBMCs from SU vs. DS; expression of CXCR3 in memory and naive γδ T cells among unstimulated PBMCs at week 104 [week 104 (PMAi stim: SU, *n* = 21, DS, *n* = 29; unstim: SU, *n* = 20, DS, *n* = 29); week 117 (PMAi stim and unstim: SU, *n* = 17, DS, *n* = 27)]. **c** Plasma CXCL9 level compared between SU (*n* = 8) and DS (*n* = 13) at week 117. In the boxplots, medians are shown, and the “hinges” represent the first and third quartile. The whiskers are the smallest and largest values after exclusion of outliers (greater than the 75th percentile plus 1.5 times the interquartile range (IQR), or less than 25th percentile minus 1.5 times the IQR). *q* values were computed using the *χ*^2^ tests in mixed-effects models, adjusted for multiple hypothesis testing using the Benjamini and Hochberg method. **d** Age of wheezing onset for SU participants with asthma (*n* = 11) was compared with that for DS participants (*n* = 15). *p* value was computed using unpaired, two-sided, non-parametric Mann–Whitney test. Source data are provided as a Source Data file.

We further probed immune differences in SU vs. DS participants by non-specifically stimulating PBMCs with PMAi. Lower frequencies of IL-4^+^ CD4^+^ memory T effector (CD45RA^-^CD127^+^CD25^lo^) cells were observed in SU compared with DS participants at week 104, which was consistent with a reduced Type 2 response (Fig. 4a; *q* = 0.02). Interestingly, lower frequencies of IFNγ^+^CD4^+^ memory T effector cells at weeks 104 and 117 (Fig. 4a; *q* = 0.07 and 0.04 respectively), and lower expression levels of IFNγ by CD4^+^ memory T effector cells (Fig. 4a; *q* = 0.06) and memory γδ T cells (Fig. 4b; *q* = 0.044) were observed in SU vs. DS participants at week 104. Unsupervised clustering analysis also corroborated lower expression levels of IFNγ by CD4^+^ memory T effector cells of SU participants compared to DS at week 117 (Supplementary Fig. 7a; *q* = 0.003). Moreover, memory and naive γδ T cells in unstimulated PBMCs displayed lower expression levels of IFNγ-inducible chemokine receptor CXCR3 at week 104 from SU vs. DS participants (Fig. 4b). Concomitantly, significantly lower levels of MIG (CXCL9), an IFN-γ-induced ligand for CXCR3, were also observed in SU compared with DS participants’ plasma at week 117 (Fig. 4c). Thus, lower expression of IFN-γ in memory γδ T cells was consistent with the lower expression of CXCR3 and reduced levels of CXCL9.

Since CD8^+^ T cells and IFN-γ have been implicated as contributing to the development of asthma^16^, we asked if the age of wheezing onset, a prelude to clinical asthma diagnosis by spirometry at the age of 7 years or older, differed in the participants that developed SU compared with DS participants. Notably, most DS participants reported wheezing onset at a significantly lower age than SU (Fig. 4d; *p* = 0.04). Age at onset of other atopic comorbidities, such as atopic dermatitis and allergic rhinitis, if present, as well as age at diagnosis of peanut allergy were comparable among SU vs. DS participants (Supplementary Fig. 7b; *p* = 0.51, 1 and 0.80, respectively).

## Discussion

Our overarching aim for this study was to understand those T-cell changes that might elucidate why SU develops only in a subset of desensitized participants following a period of allergen withdrawal. Using agnostic computer-based learning algorithms as well as hypothesis-based analyses from high dimensional immune studies, our findings suggest a pivotal function of CD8^+^ T cell differentiation status in defining the likelihood of SU. Our findings suggest that SU is favored by: (i) a lower frequency of terminally differentiated, CD57^+^ allergen-reactive, and allergen non-reactive CD8^+^ T cells possibly coupled with a higher frequency of cytotoxic EM CD8^+^ T cells, (ii) a lower frequency of naive CD8^+^ T cells, (iii) a lower expression of CD86 on APCs and/or a lower frequency of CD86^+^ APCs pre-OIT, and (iv) a lower frequency of IFN-γ^+^CD4^+^ memory T effectors post-OIT.

While many clinical studies have confirmed the efficacy of peanut OIT in conferring desensitization, only a handful have tested the induction of SU during the post-avoidance period. Moreover, the immune mechanistic studies performed during a subset of such SU testing trials primarily have been focused on serological readouts (i.e., peanut-specific IgE and IgG4), basophil activation, measurement of Th2 cytokines, and changes in the frequency of total and peanut-reactive CD4^+^ Tregs and Teffectors^14,17–20^. A recent study examining longitudinal transcriptional changes and clonality of peanut-reactive Th cell subsets from peanut OIT participants offers further refined insights into OIT-induced changes and features predictive of post-OIT outcome in the context of CD4^+^ T cells^21^. However, a comprehensive evaluation of circulating immune cell frequencies and functional markers, and the cumulative potential impact of any significant changes distinguishing SU in a given OIT cohort, has not been previously performed. Our analyses put forth such an evaluation by employing high dimensional, multi-parametric tools such as mass cytometry and Luminex. Our study thus provides unique insights into immune players in the induction of OIT-induced tolerance.

Notably, we observed significant differences beyond Th2 and Treg subsets while comparing immunophenotypes of SU vs. DS participants within our CyTOF dataset. In agreement with previous reports, attenuation of Th2 phenotype was observed exclusively among active participants post-OIT compared to placebo-treated participants, while no change was noted in the frequencies of pr CD4^+^ T cells and Tregs post-OIT^17,21^. Interestingly, in addition to prototypical Th2 cytokines, the frequency of a regulatory cytokine- IL-10^+^ pr CD4^+^ T cells was seen to be diminished in active participants post-OIT. This might be attributed to an initial autocrine IL-10 induction in pathogenic pr CD4^+^ T cells in response to peanut dose build-up followed by a marked decrease by week 104 post-OIT^22^. However, CD4^+^ T cell subset readouts did not significantly distinguish SU vs. DS in our study or those by others^14,17^. Instead, higher frequencies of naive CD8^+^ and lower frequencies of EM CD8^+^ T cells, at BL and throughout the course of OIT, identified those individuals with less likelihood of becoming SU. Our preliminary data demonstrate that CD8^+^ T cells from SU participants have higher GzB-mediated cytolytic potential. Of note, GzB has been shown to be critical for TCR-induced death of Th2 cells^23,24^ thus raising a possibility of EM CD8^+^ cell sourced-GzB conferring SU participants a selective advantage by regulating pathogenic peanut-reactive Th2 proliferation, and thus peanut-specific IgE during avoidance. The significant negative correlation observed between the BL frequency of EM CD8^+^ cells and peanut-specific as well as Ara h 2-specific IgE reinforces this possibility. Investigations are underway to evaluate how GzB from EM CD8^+^ cells may preferentially target immune cell subsets in the IgE pathway, and influence of CD57 expression on GzB function in this context. The higher frequency of naive CD8^+^ T cells, despite higher expression of CD86 on APC subsets and a higher level of IL-2 in peanut-stimulated PBMC culture supernatants, observed in DS compared to SU participants at BL likely implies a higher threshold of activation. Of note, our group has recently reported hypomethylation of the IL-2 locus, and consequently higher levels of IL-2, in peanut-stimulated PBMC culture supernatants as one epigenetic readout distinguishing peanut-allergic twins from their non-allergic counterparts^25^.

Concomitantly, a higher frequency and expression of CD57 on EM and TEMRA CD8^+^ T cells in DS participants suggests terminal differentiation and immunosenescence. Early childhood viral exposure, chronic in vivo inflammation, and natural aging may drive such terminal differentiation. The speculation regarding early childhood viral exposure also is consistent with the significantly lower age of wheezing onset we diagnosed for DS participants (Fig. 4d).

CD57 and CD27 have reciprocal expression patterns in T cell ontology^26^. Interestingly, terminally differentiated CD27^-^CRTH2^+^CD161^+^ memory allergen-reactive CD4^+^ T cells, termed Th2A cells, have been strongly associated with allergies^27^. Despite being frequently associated with senescence, CD57^+^ CD8^+^ T cells subsets have been reported to be highly proinflammatory^26^. We also observed a higher frequency of IFNγ^+^CD4^+^ T effectors among post-OIT PBMCs of DS participants on PMA/Ionomycin stimulation. Thus, taken together, preexistence of terminally differentiated, proinflammatory CD8^+^ and CD4^+^ subsets compounded with a high-activation-threshold, “reluctant-to-differentiate” CD8^+^ T cells in DS participants, likely pose immune plasticity challenges. These challenges might be mitigated during active dosing of OIT leading to successful desensitization, although they can resurface post-avoidance, and are reflected through clinical relapse/reactivity to allergens.

There are limitations to our study. Firstly, not all immune subsets of interest such as Tfh subsets^21,28^, Th2a^27^ cells could be monitored through our CyTOF panel. Secondly, a thorough longitudinal analysis inclusive of PBMCs and plasma from additional time points over the course of OIT was not feasible due to limited sample availability. In addition, unlike manual gating, FlowSOM-based unsupervised clustering analysis could not identify certain rare immune cell subsets as clusters, particularly pr CD8^+^ T cells. Semi-supervised cell type annotation algorithm- CyAnno^29^ developed by our group will likely yield a better output in this regard. In addition, the ML models used to identify features for classification of SU vs. DS groups were based on small sample size of training and testing datasets, which may have led to model over-fitting, and would be a major limitation of models proposed in this study and features identified. To address this issue, we simulated the real dataset 300 times and built elastic net models for each of them. Here, the elastic net model was applied because it uses L1 and L2 regularization penalties for model building and the algorithm has been proven to be useful for similar cases where the number of features are larger than the number of samples. To further minimize the effect of model over-fitting, we only prioritized the SU vs. DS classification features that are conserved across 300 elastic net model. The current ML model based findings nonetheless warrant validation using a similar, independent dataset of food OIT study participants tested for SU.

Higher levels of allergen- or allergen-component-specific IgE pre-OIT have been reported in the past to predict a negative outcome towards SU^13,14^. Frequency of naive CD8^+^ T cells positively correlated with peanut and Ara h 2-specific IgE, while abundance of CD57^+^ pr CD8^+^ T cells negatively correlated with the Ara h 2-specific IgG/IgE ratio at BL. Thus, the CD8^+^ T cell differentiation status, along with the frequency of IL-4^+^ and IL-9^+^ pr CD4^+^ T cells, may be considered as potential biomarkers predictive of SU following OIT. Prospective peanut-allergic participants presenting with such skewed CD8^+^ T cell differentiation may benefit from continued peanut dosing. In addition, adjunctive treatment with anti-IgE, which has been shown to reduce the frequency of CXCR3^+^ CD8^+^ T cells^30^ could be helpful in certain patients. Moreover, future research to examine the function of CD8^+^ T cell subsets in the context of food allergy pathogenesis and SU needs to be extended to include regulatory CD8^+^ T cells^31^ and Tc2 subpopulations^32^, the identification of which was beyond the scope of our CyTOF panel.

The CyTOF and Luminex data presented here confirmed attenuation of Th2 phenotype as well as reduction in proinflammatory cytokines in active participants at week 104 post-OIT. In addition, the Luminex data also showed significant changes in monocyte and DC differentiation-related mediators such as GCSF, GMCSF and FLT3L, as well as a reduction in tissue homeostasis factors viz. IL-22 and VEGF, which warrant further investigation.

Taken together, this study demonstrates that frequencies of non-Th2 immune cells, primarily CD8^+^ T cell subsets, may dictate the likelihood of achieving sustained unresponsiveness following food allergy OIT.

## Methods

### Study participants

The clinical research protocol for POISED study was approved by the Division of Allergy, Immunology, and Transplantation (DAIT) and the National Institute of Allergy and Infectious Diseases (NIAID) Allergy and Asthma Data Safety Management Board, the DAIT/NIAID Clinical Review Committee, the Stanford Institutional Review Board, and the US Food and Drug Administration (FDA). Written informed consent was obtained from adult participants or parents/ guardians of minor participants along with assent from minor participants of age 7 years and older. All compliant participants were given $30 gift cards as a compensation for each study visit as consistent with Stanford IRB-approved protocol. The study protocol, patient demographics, and treatment outcomes have been described in detail previously^8^. Briefly 120 peanut-allergic participants (age: 7–55 years) were randomized into active vs. placebo group. Participants in the active arm (*n* = 95) were built up and maintained on 4000 mg peanut protein through week 104. Fifty-one of the total per-protocol active arm participants were randomized to discontinue peanut consumption post-week 104 (peanut-0 group). DBPCFCs to 4000 mg peanut protein were done at BL and weeks 104, 117, 130, 143, and 156. Participants in peanut-0 group, who passed oral food challenge (OFC) at week 117 (i.e., Peanut-0 Successes) were denoted as “Sustained unresponsive”, while those, who failed week 117 OFC (i.e., Peanut-0 Failures) were termed as “Desensitized”. Baseline demographics for all the participants in the study have been previously published^8^. Supplementary Table 1 shows baseline demographic characteristics for the participants per randomization (i.e., active vs. placebo) and outcome (i.e., SU vs. DS), and for the subset, whose samples were analyzed through Luminex.

### Blood draws and processing

From each study participant, ~40 mL blood was drawn by venipuncture and collected in EDTA tubes at baseline (BL, i.e., during the initial screening phase or at week 0 post-enrollment), and every 3 months over the course of treatment. PBMCs and plasma isolated by Ficoll-based density gradient centrifugation were frozen in aliquots, and stored in liquid nitrogen at −80 °C, until thawing for respective assays. In these analyses, we used PBMCs and plasma stored at BL, week 104, and week 117.

### Mass cytometry

Mass cytometry studies were performed on PBMC samples at BL, week 104, and week 117 as listed in Supplementary Table 2. PBMCs frozen per time point per participant were thawed and rested overnight at 37 °C with 5% CO_2_ in RPMI + 10% FBS, and Pen-Strep. Cells were counted, and plated in 3 round-bottom wells of a 96-well plate at the density of 3 × 10^6^ cells in culture with 300 μL RPMI, 5% FBS, Pen-Strep per well. To evaluate the allergen (peanut)-induced response, PBMCs in one of the wells were stimulated with 200 μg/mL peanut solution for 24 h with the addition of brefeldin A (5 μg/mL; BioLegend, San Diego, CA) for the last 4 h. PBMCs in the second well were left unstimulated, treated with brefeldin A for 4 h before harvesting and served as controls. PBMCs in the third well were stimulated with 20 ng/mL PMA + 1 μg/mL Ionomycin (Sigma-Aldrich) for 4 h in the presence of brefeldin A to measure cytokine expression. The downstream steps comprising cell harvesting, staining, were followed as previously described^12^. Briefly, the cells were harvested at the end of the incubation period, washed with CyFACS buffer (0.1% bovine serum albumin, 0.1% sodium azide, 2 mM EDTA in PBS), and stained with surface antibody cocktail (Supplementary Data 1) for 30 min at room temperature in dark followed by staining with live/dead 115-DOTA maleimide. The cells were then washed with CyFACS buffer and barcoded using Cell-ID 20-Plex Pd Barcoding Kit (Fluidigm Co., Product# 201060) according to the manufacturer’s instructions. Barcoded cells were fixed with 2% paraformaldehyde overnight at 4 °C. Following a CyFACS wash the next day, equal number of cells per sample were pooled, permeabilized, and stained with intracellular antibody cocktail (Supplementary Data 1) for 30 min at room temperature. The cells were then washed twice with permeabilization buffer followed by two washes with CyFACS buffer. Finally, the pooled samples were stained with Ir interchelator, washed twice in CyFACS buffer, followed by one wash in CyPBS and two further washes in MilliQ water. Approximately 300,000 cells per sample in each pool were acquired on a Helios mass cytometer (Fluidigm Co.). Data normalization, concatenation and debarcoding were performed using CyTOF software (Fluidigm Co.). Data were analyzed by manual gating as well as FlowSOM-based unsupervised clustering analysis.

Manual gating on normalized, de-barcoded data files was carried out using FlowJo v10 (FlowJo LLC., Ashland, OR). Gating scheme used to identify and analyze CD3^+^ and CD3^-^ immune cell subsets has been previously illustrated^30^ as well as depicted in Supplementary Fig. 8a, b. Workflow illustrations were generated using BioRender application.

The FlowSOM-based unsupervised computational analysis was performed separately for peanut-stimulated, unstimulated, and PMA/ionomycin-stimulated samples at independent time points (i.e., week 0, week 104, and week 117)^33^. CD127 was excluded to minimize batch effects, and the remaining lineage markers were transformed using arcsinh function (inverse hyperbolic sine; cofactor of 5), followed by their further normalization with landmark alignment procedure using the warpSet function from the flowStats R package (version 4.4.0)^34^. In each analysis 50,000 live cells were randomly selected and subjected to FlowSOM-based clustering with default parameters and with a fixed number of 30 clusters. A defined set of lineage markers were used for cell clustering (Supplementary Table 3). Distinct clusters represented as a heatmap (R package pheatmap, version 1.0.12)^35^ were manually annotated by visual inspection of median expression of lineage markers per cluster. In addition, 100,000 cells were randomly selected from the predicted clusters for further visualization (R package ggplot2 version 3.3.3)^36^ by applying the non-linear dimensionality reduction technique- Uniform Manifold Approximation and Projection (UMAP) at the lineage marker levels using the R package UMAP (version 0.2.7.0, default parameters except min_dist = 0.25)^37^.

Significant differences in frequency (i.e., proportion or abundance within a given parent population) of cell subsets and median intensity (i.e., expression) of functional markers within a cell subset were evaluated by manual gating as well as unsupervised clustering analysis. For both these approaches, *p* values were computed using the *χ*^2^ tests in mixed-effects models, wherein defined groups were used as fixed-effects and batch number as random-effects using lmerTest R package^38^. The computed *p* values were adjusted to control the FDR during multiple hypothesis testing using the Benjamini and Hochberg method. Readouts with *p* value < 0.05 and adjusted *p* value (i.e., *Q* value) <0.1 have been reported as significant in this analysis. Any extreme outlier data point(s) were identified and removed using identify_outliers() function (coef = 3.0) from rstatix R package (Version 0.7.0)^39^.

### Luminex assay

Luminex analysis was performed on a convenience sample subset (Supplementary Table 1). PBMCs were thawed, rested for 6 h, and plated in 2 round-bottom wells of a 96-well plate at the density of 1.4 × 10^6^ cells in culture with 200 μL RPMI, 5% FBS, Pen-Strep per well. PBMCs in one of the wells were stimulated with 200 μg/mL peanut solution for 18 h, while the ones in the other well were left unstimulated for that duration. A total of 130 μL culture supernatant was harvested per well in prelabeled microfuge tubes and stored at −80 °C until performing the assay. Culture supernatants were probed with Human 48-plex Cytokine/Chemokine magnetic bead panel, while plasma samples were tested using a combination of 48-plex and 23-plex Cytokine/Chemokine magnetic bead panel (MilliporeSigma, Burlington, MA). While comparing the readouts (MFIs), *p* values were computed using Wilcoxon signed-rank sum test (unpaired and paired where Time is used as group), and adjusted for multiple hypothesis testing using Benjamini and Hochberg method. The rstatix R package was used to identify and remove any extreme outlier data point(s)^39^.

### Evaluation of Granzyme B expression by flow cytometry

Week 52 PBMCs from 5 SU and DS participants each were thawed and rested overnight. Approximately 0.3 million PBMCs were stimulated with 20 ng/mL PMA and 1 μg/mL Ionomycin for 4 h, and equivalent number of PBMCs per participant was left unstimulated. Brefeldin A (5 μg/mL) and Monensin (2 μM) was added to PBMCs during PMAi stimulation as well as to unstimulated PBMCs for 4 h prior to harvesting. On harvesting, cells were stained with antibodies listed in Supplementary Data 1. Data were acquired on CyTek Dx10 flow cytometer and analyzed using FlowJo v10 (FlowJo LLC., Ashland, OR).

### Spearman correlations

Spearman correlations between mass cytometry and serological parameters were performed for SU and DS participants. The correlation coefficient (Rho; *p*) and associated *p* value significance was computed using cor.test() R function. Correlations with ǀ*p*ǀ > 0.4 and *p* < 0.001 were considered as significant and were shortlisted for subsequent evaluation. The resulting correlations were plotted using ggplot R function and linear regression line with 95% confidence interval shading was computed using geom_smooth() R function (method = *lm*).

### Machine learning analysis

To identify the BL mass cytometry (CyTOF) parameters that distinguish SU participants from DS, we applied a Machine learning (ML) approach on BL simulated datasets (*n* = 300; see Supplementary Fig. 6a, b). Data simulation was used to compensate for the small sample size (*n* = 46 at BL) available for model training as well as to obtain confidence intervals for model performance evaluation, using the simstudy R package^40^. Here, the data were simulated (300 times) from manually gated frequencies and marker expression of cell types from BL CyTOF samples with a set of constraints, including sample size, mean, and variance per participant *p*, i.e., *X*_*p*_ ~ *N*(μ,σ^2^). Each of the simulated dataset had the same sample size as the original BL dataset with overall same mean and variance of CyTOF parameters in the respective participant (i.e., 46 samples and 123 CyTOF parameters). For ML prediction, we used elastic net algorithm which is a regularized logistic regression method that uses a linear and weighted combination of L1 and L2 regularization of the lasso and ridge methods^41^. This ML algorithm is considered as suitable for the datasets wherein there are more features (123 features) than instances (i.e., 46 participants). Here, the *glmnet* R package (version 4.1-2) was used for executing elastic net, wherein the model parameters were tuned using k-fold cross-validation^42^. Finally, each of the given simulated dataset was used for building elastic net model and the feature importance matrix were computed for respective dataset. The model accuracy was evaluated by comparing the predicted outcome with expected outcome, and after 300 runs, the performance statistics, including specificity, sensitivity, and area under the curve (AUC) were also evaluated. Robust CyTOF parameters in each elastic net model were then defined as those features with score greater than 80 (arbitrary selected threshold; max(score) = 100 and min(score) = 0). The cumulative importance score for each feature was defined as:

1 $${{{{{\rm{Cumulative}}}}}}\,{{{{{\rm{importance}}}}}}=\frac{{\sum }_{i=1}^{i={{{{{\rm{iterations}}}}}}}\delta }{{{{{{\rm{iterations}}}}}}}\times\,100$$

Where, iteration is the number of times (=300) simulated datasets were modeled, and *δ* is the feature score computed for each model, and its value in each iteration *i* is defined as:

2 $$\delta=\left\{\begin{array}{cc}1,& {{{{{{\rm{if}}}}}}}\,{\delta }_{i},\, > \,80\\ 1,& {{{{{{\rm{if}}}}}}}\,{\delta }_{i}0,\,\le \,80\end{array}\right.$$

### Reporting summary

Further information on research design is available in the Nature Research Reporting Summary linked to this article.

## Supplementary information

Supplementary Information

Description of Additional Supplementary Files

Supplementary Data 1

Reporting Summary

## Data Availability

The raw unlabeled POISED Mass Cytometry dataset files generated in this study in FCS 3.0 format, associated metadata, and the gating schema have been deposited in the FlowRepository database under accession codes FR-FCM-Z4MA and FR-FCM-Z2V9. The processed CSV files are provided in the Source Data file, which is available on the dryad digital repository under the DOI accession 10.5061/dryad.95x69p8p6. Source Data are provided with this paper.

## Source data

Source Data
