# Supplementary material for: CD8+ T cell differentiation status correlates with the feasibility of sustained unresponsiveness following oral immunotherapy
Source: Nat Commun. 2022 Nov 4;13:6646. doi: 10.1038/s41467-022-34222-8 (PMC9636180; doi:10.1038/s41467-022-34222-8)
Supplement: Supplementary file 1 — Supplementary Information [file 41467_2022_34222_MOESM1_ESM.pdf]

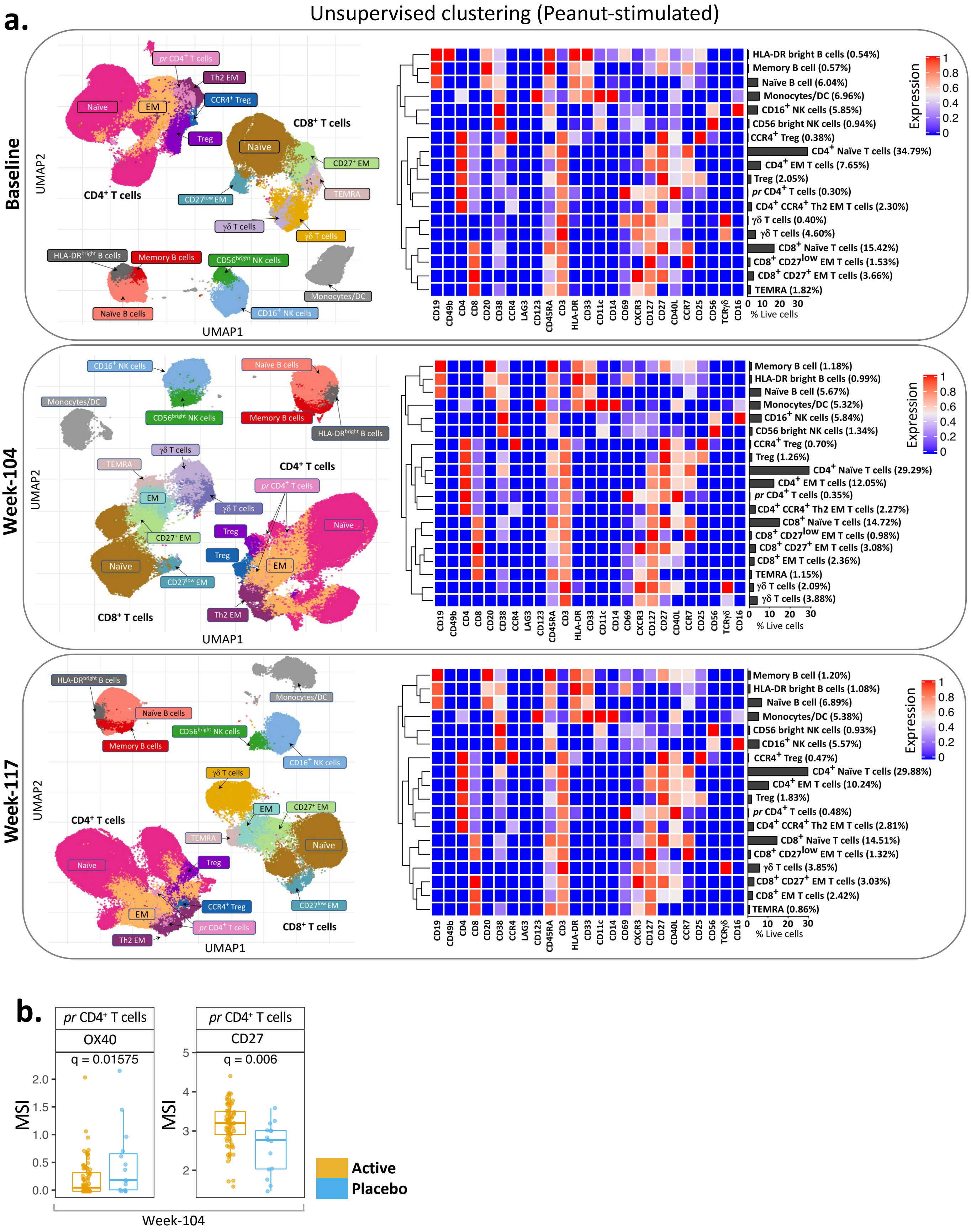
a.
Unsupervised clustering (Peanut-stimulated)
Baseline
Week-104
Week-117
UMAP1
UMAP2
CD4+ T cells
CD8+ T cells
Expression
% Live cells
HLA-DR bright B cells (0.54%)
Memory B cell (0.57%)
Naïve B cell (6.04%)
Monocytes/DC (6.96%)
CD16+ NK cells (5.85%)
CD56 bright NK cells (0.94%)
CCR4+ Treg (0.38%)
CD4+ Naïve T cells (34.79%)
CD4+ EM T cells (7.65%)
Treg (2.05%)
pr CD4+ T cells (0.30%)
CD4+ CCR4+ Th2 EM T cells (2.30%)
γδ T cells (0.40%)
γδ T cells (4.60%)
CD8+ Naïve T cells (15.42%)
CD8+ CD27low EM T cells (1.53%)
CD8+ CD27+ EM T cells (3.66%)
TEMRA (1.82%)
Memory B cell (1.18%)
HLA-DR bright B cells (0.99%)
Naïve B cell (5.67%)
Monocytes/DC (5.32%)
CD16+ NK cells (5.84%)
CD56 bright NK cells (1.34%)
CCR4+ Treg (0.70%)
Treg (1.26%)
CD4+ Naïve T cells (29.29%)
CD4+ EM T cells (12.05%)
pr CD4+ T cells (0.35%)
CD4+ CCR4+ Th2 EM T cells (2.27%)
CD8+ Naïve T cells (14.72%)
CD8+ CD27low EM T cells (0.98%)
CD8+ CD27+ EM T cells (3.08%)
CD8+ EM T cells (2.36%)
TEMRA (1.15%)
γδ T cells (2.09%)
γδ T cells (3.88%)
Memory B cell (1.20%)
HLA-DR bright B cells (1.08%)
Naïve B cell (6.89%)
Monocytes/DC (5.38%)
CD56 bright NK cells (0.93%)
CD16+ NK cells (5.57%)
CCR4+ Treg (0.47%)
CD4+ Naïve T cells (29.88%)
CD4+ EM T cells (10.24%)
Treg (1.83%)
pr CD4+ T cells (0.48%)
CD4+ CCR4+ Th2 EM T cells (2.81%)
CD8+ Naïve T cells (14.51%)
CD8+ CD27low EM T cells (1.32%)
γδ T cells (3.85%)
CD8+ CD27+ EM T cells (3.03%)
CD8+ EM T cells (2.42%)
TEMRA (0.86%)
CD19 CD49b CD4 CD8 CD20 CD38 CCR4 LAG3 CD123 CD45RA CD3 HLA-DR CD33 CD11c CD14 CD69 CXCR3 CD127 CD27 CD40L CCR7 CD25 CD56 TCRγδ CD16
b.
pr CD4+ T cells
OX40
q = 0.01575
CD27
q = 0.006
MSI
Active
Placebo
Week-104

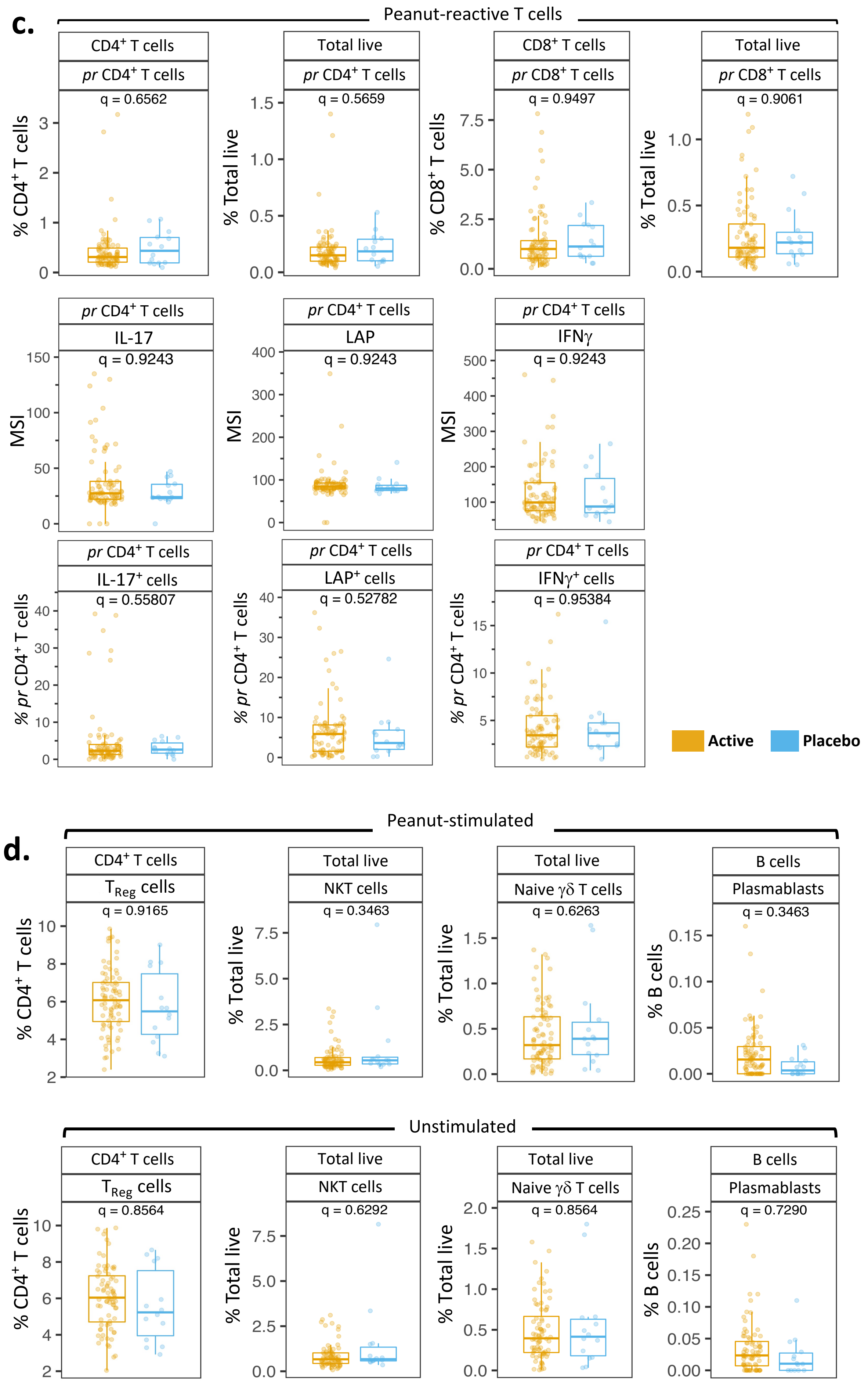
c.
Peanut-reactive T cells
CD4+ T cells
pr CD4+ T cells
q = 0.6562
% CD4+ T cells
Total live
pr CD4+ T cells
q = 0.5659
% Total live
CD8+ T cells
pr CD8+ T cells
q = 0.9497
% CD8+ T cells
Total live
pr CD8+ T cells
q = 0.9061
% Total live
pr CD4+ T cells
IL-17
q = 0.9243
MSI
pr CD4+ T cells
LAP
q = 0.9243
MSI
pr CD4+ T cells
IFNγ
q = 0.9243
MSI
pr CD4+ T cells
IL-17+ cells
q = 0.55807
% pr CD4+ T cells
pr CD4+ T cells
LAP+ cells
q = 0.52782
% pr CD4+ T cells
pr CD4+ T cells
IFNγ+ cells
q = 0.95384
% pr CD4+ T cells
Active
Placebo
d.
Peanut-stimulated
CD4+ T cells
TReg cells
q = 0.9165
% CD4+ T cells
Total live
NKT cells
q = 0.3463
% Total live
Total live
Naive γδ T cells
q = 0.6263
% Total live
B cells
Plasmablasts
q = 0.3463
% B cells
Unstimulated
CD4+ T cells
TReg cells
q = 0.8564
% CD4+ T cells
Total live
NKT cells
q = 0.6292
% Total live
Total live
Naive γδ T cells
q = 0.8564
% Total live
B cells
Plasmablasts
q = 0.7290
% B cells

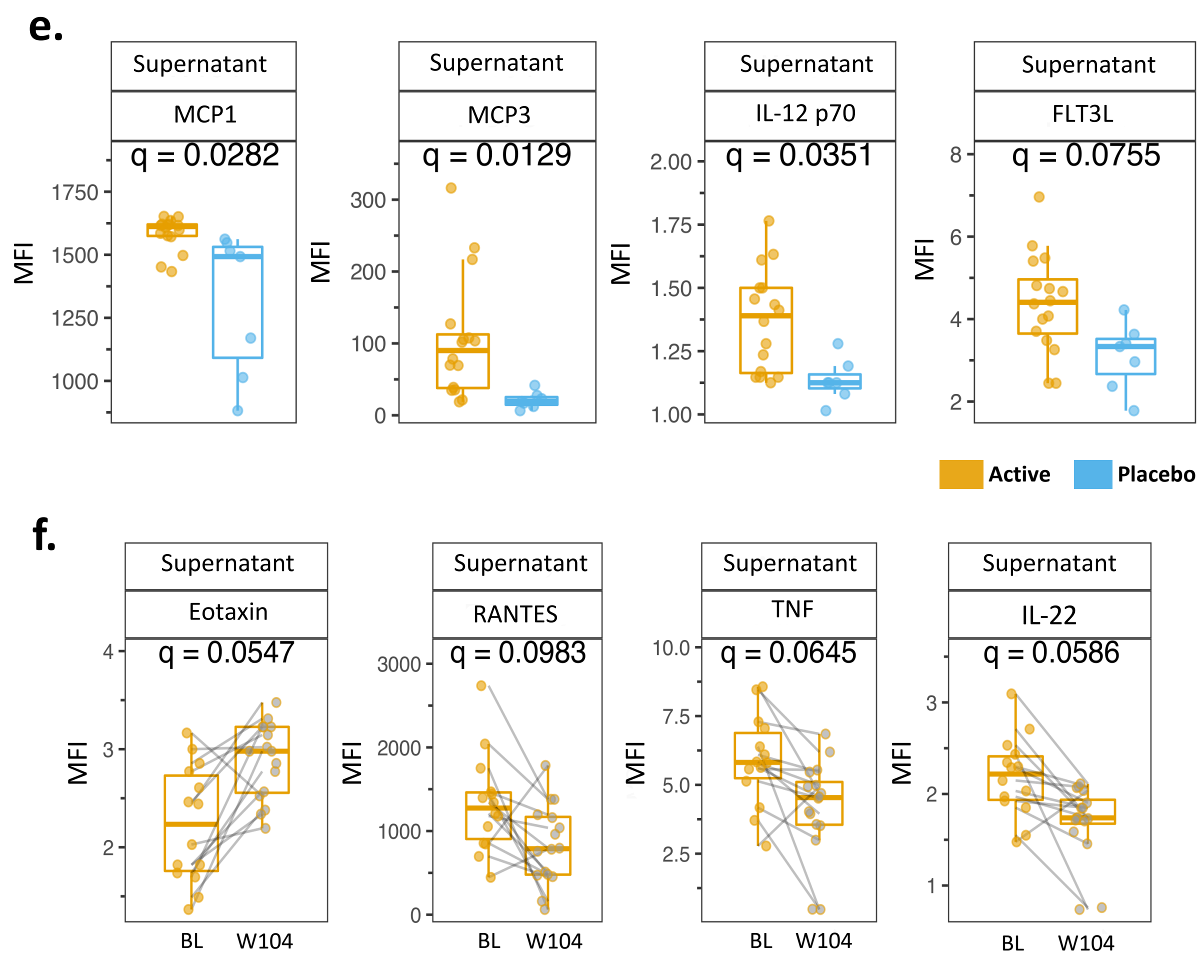

Supplementary Fig. 1: Peanut OIT induces attenuation of the Th2 phenotype. a, Uniform Manifold Approximation and Projection (UMAP) depicting distinct immune cell subsets identified using FlowSOM- based unsupervised clustering of peanut-stimulated BL, week 104, week 117 PBMCs. Clusters annotated as distinct immune cell subsets among peanut-stimulated week 104 PBMCs from participants on active peanut OIT (n = 80) were compared against those treated with placebo (n = 15). **b**, Significant changes in the expression of OX40 and CD27 in peanut-reactive CD4^{+} T cell cluster cross-verified using manual gating are shown. [Week 104 (Peanut stim: Active, n = 80; Placebo, n = 15)] . **c,d,** Non-significant changes in marker expression and frequency within pr CD4^{+} and pr CD8^{+} T cells among peanut-stimulated [Week 104 (Peanut stim: Active, n = 80; Placebo, n = 15)] (**c**), and non-peanut reactive immune cell subsets among peanut-stimulated and unstimulated PBMCs (**d**). **e,f,** Significant changes in peanut-stimulated week 104 PBMC culture supernatant of active participants (n = 17), compared to those treated with placebo (n = 7) (**e**), and compared to BL (**f**). In the box-plots, medians are shown, and the 'hinges' represent the first and third quartiles. The whiskers are the smallest and largest values after exclusion of outliers (greater than the 75th percentile plus 1.5 times the interquartile range (IQR), or less than 25th percentile minus 1.5 times the IQR). Q values were computed using the χ^2-test in mixed-effects models, adjusted for multiple hypothesis testing using the Benjamini and Hochberg method. Source data are provided as a Source Data file.

a.

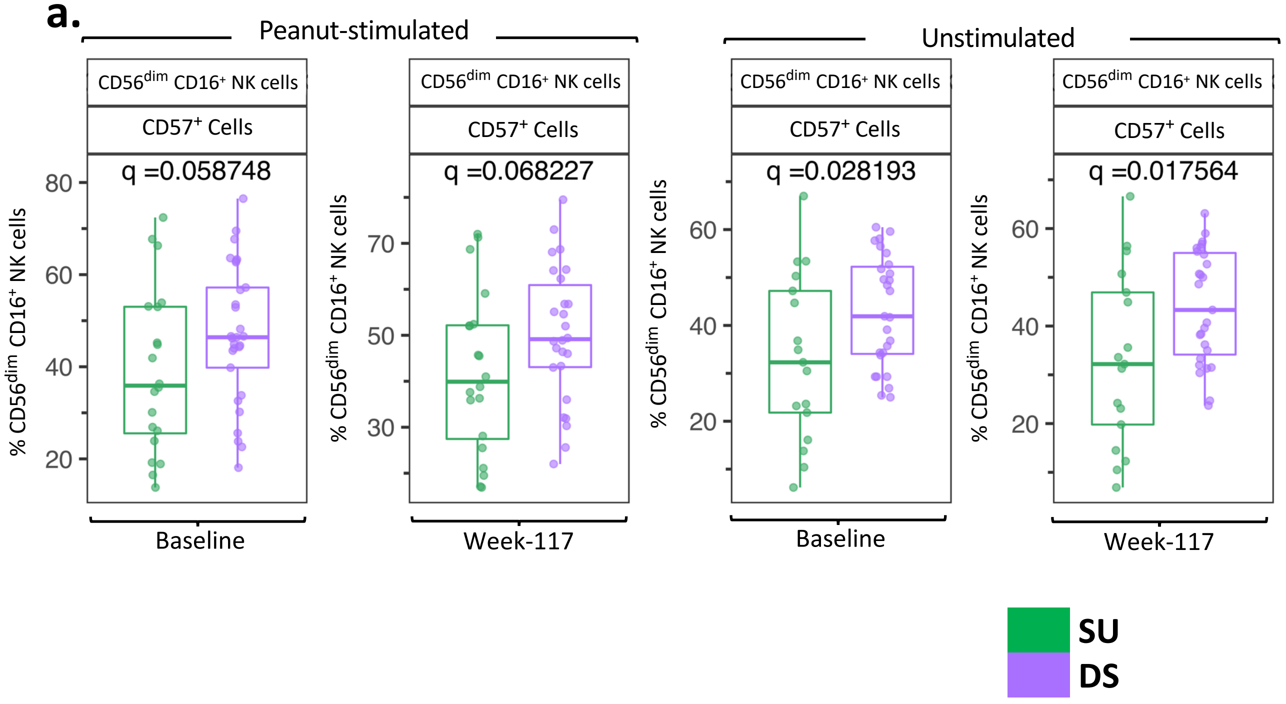
Peanut-stimulated
Unstimulated
CD56dim CD16+ NK cells
CD57+ Cells
q =0.058748
q =0.068227
q =0.028193
q =0.017564
% CD56dim CD16+ NK cells
Baseline
Week-117
SU
DS

Supplementary Figure 2

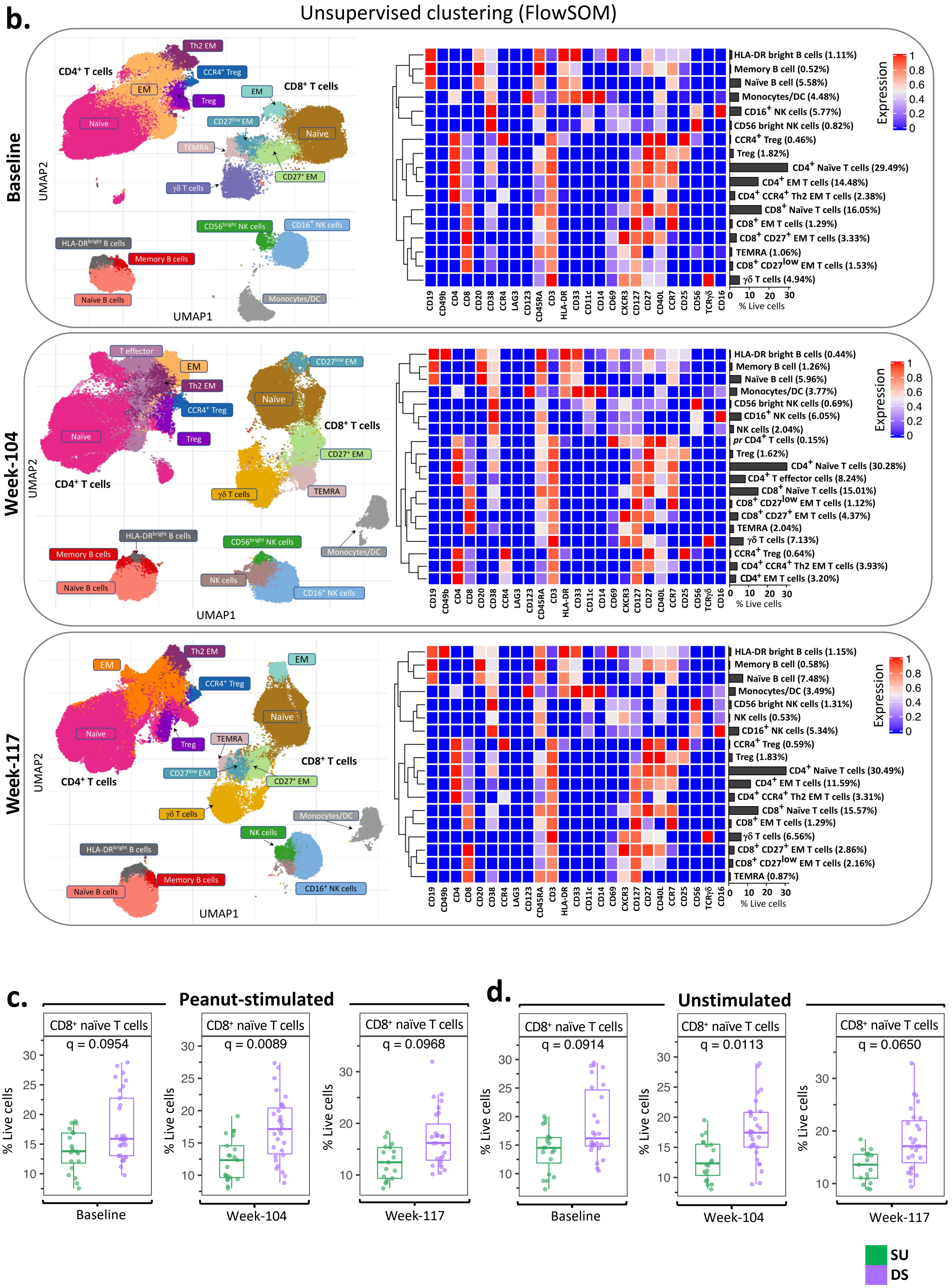

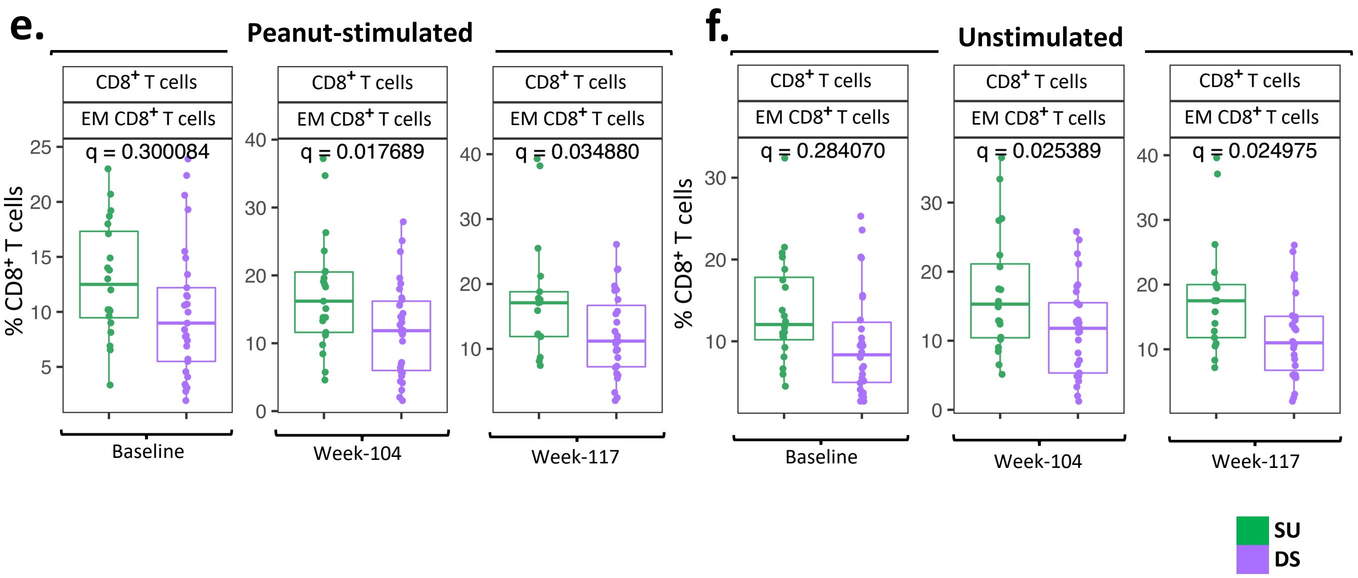

Supplementary Fig. 2: Higher CD57^{+} CD56^{dim} CD16^{+} NK cell and higher naïve CD8^{+} vis-à-vis lower EM CD8^{+} T cell frequencies discern DS participants from SU. a, Higher frequencies of CD57^{+}CD56^{dim}CD16^{+} NK cell in DS participants compared to SU at BL and week 117 in peanut-stimulated and unstimulated PBMCs. [BL (pea stim: SU, n = 20, DS, n = 29; unstim: SU, n = 20, DS, n = 26), week 117 (peanut stimulated: SU, n = 17, DS, n = 27; unstim: SU, n = 17, DS, n = 27)]. **b,** UMAP depicting distinct immune cell subsets identified using FlowSOM-based unsupervised clustering of unstimulated BL, week 104, week 117 PBMCs. Frequency and marker expression on immune cell clusters among unstimulated PBMCs from SU participants were respectively compared with those from DS participants. **c,d,** Significant change in the abundance of naïve CD8^{+} T cell cluster per time point cross-verified using manual gating of peanut- stimulated **(c)** and unstimulated **(d)** PBMCs at BL, week 104 (pea stim: SU, n = 21, DS, n = 30; unstim: SU, n = 20, DS, n = 29), and week 117 are shown. **e,f,** Frequency of EM CD8^{+} T cells among total CD8^{+} T cells at BL, week 104, and week 117 in peanut-stimulated **(e)** and unstimulated **(f)** PBMCs estimated by manual gating. In the box-plots, medians are shown, and the 'hinges' represent the first and third quartiles. The whiskers are the smallest and largest values after exclusion of outliers (greater than the 75th percentile plus 1.5 times the interquartile range (IQR), or less than 25th percentile minus 1.5 times the IQR. Q values were computed using the χ^2-test in mixed-effects models, adjusted for multiple hypothesis testing using the Benjamini and Hochberg method. Source data are provided as a Source Data file.

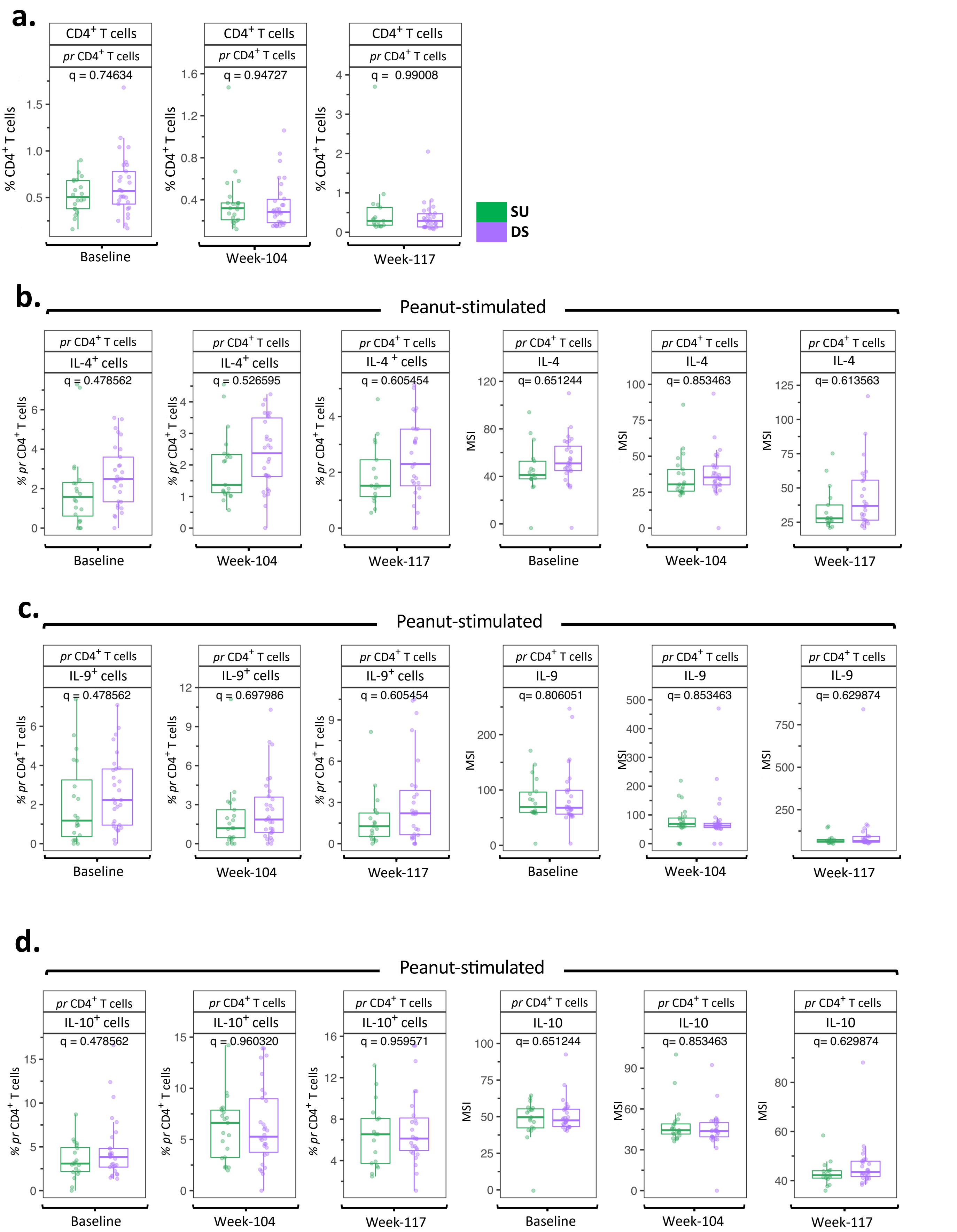
a.
b.
c.
d.
SU
DS
Peanut-stimulated
CD4+ T cells
pr CD4+ T cells
q = 0.74634
q = 0.94727
q = 0.99008
% CD4+ T cells
IL-4+ cells
q = 0.478562
q = 0.526595
q = 0.605454
IL-4
q= 0.651244
q= 0.853463
q= 0.613563
IL-9+ cells
q = 0.478562
q = 0.697986
q = 0.605454
IL-9
q= 0.806051
q= 0.853463
q= 0.629874
IL-10+ cells
q = 0.478562
q = 0.960320
q = 0.959571
IL-10
q= 0.651244
q= 0.853463
q= 0.629874
% pr CD4+ T cells
MSI
Baseline
Week-104
Week-117

e.

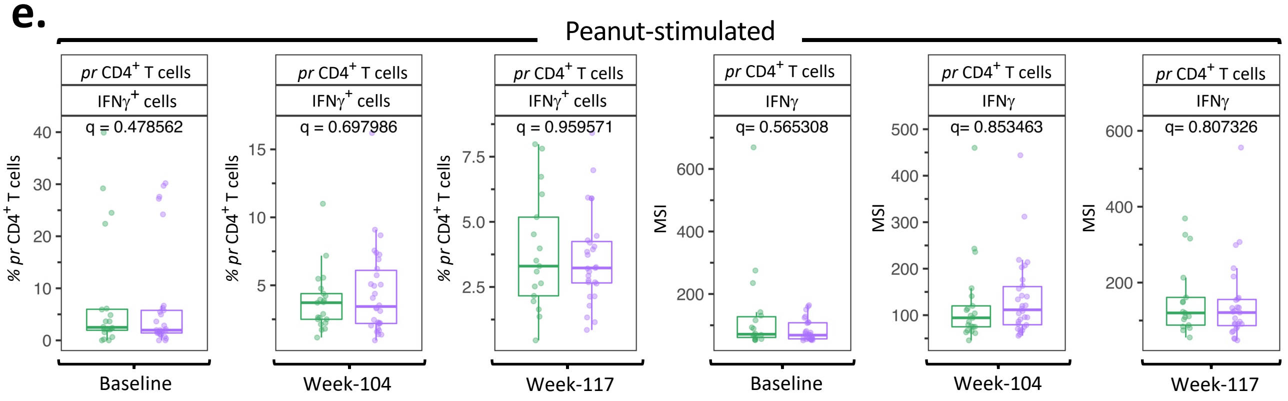
Peanut-stimulated
pr CD4+ T cells
IFNγ+ cells
q = 0.478562
q = 0.697986
q = 0.959571
IFNγ
q= 0.565308
q= 0.853463
q= 0.807326
% pr CD4+ T cells
MSI
Baseline
Week-104
Week-117

f.

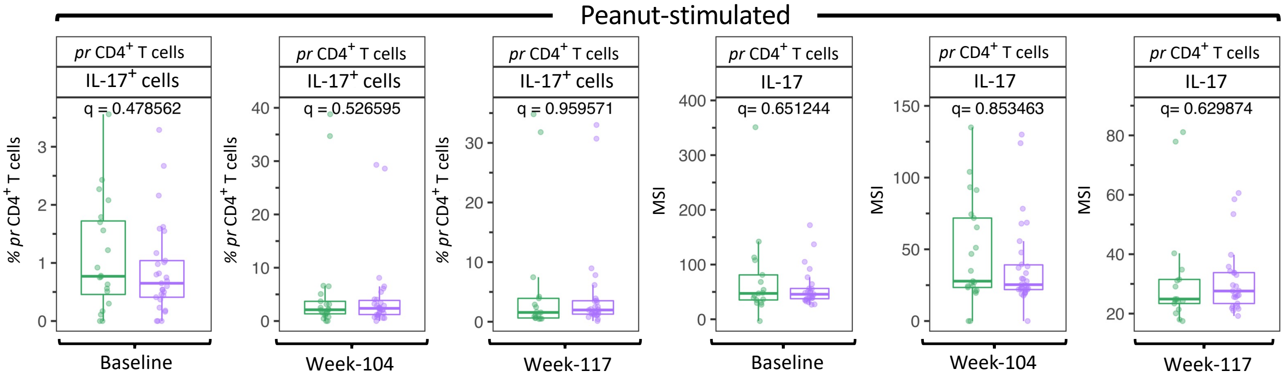
Peanut-stimulated
pr CD4+ T cells
IL-17+ cells
q = 0.478562
q = 0.526595
q = 0.959571
IL-17
q= 0.651244
q= 0.853463
q= 0.629874
% pr CD4+ T cells
MSI
Baseline
Week-104
Week-117

g.

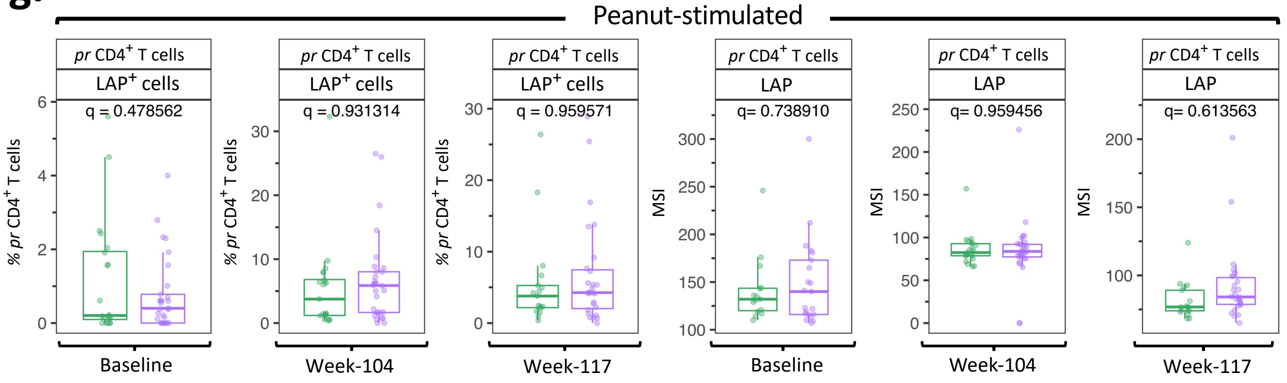
Peanut-stimulated
pr CD4+ T cells
LAP+ cells
q = 0.478562
q = 0.931314
q = 0.959571
LAP
q= 0.738910
q= 0.959456
q= 0.613563
% pr CD4+ T cells
MSI
Baseline
Week-104
Week-117

h.

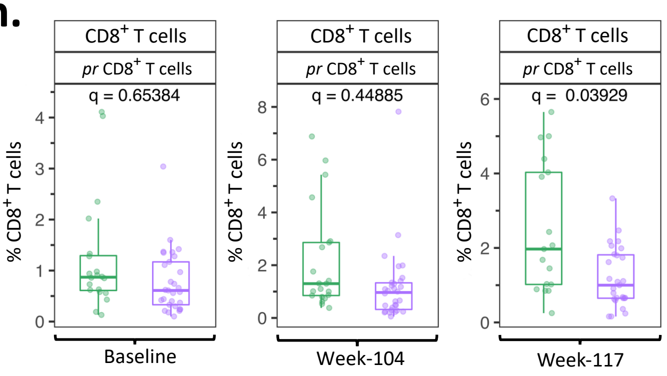
CD8+ T cells
pr CD8+ T cells
q = 0.65384
q = 0.44885
q = 0.03929
% CD8+ T cells
Baseline
Week-104
Week-117

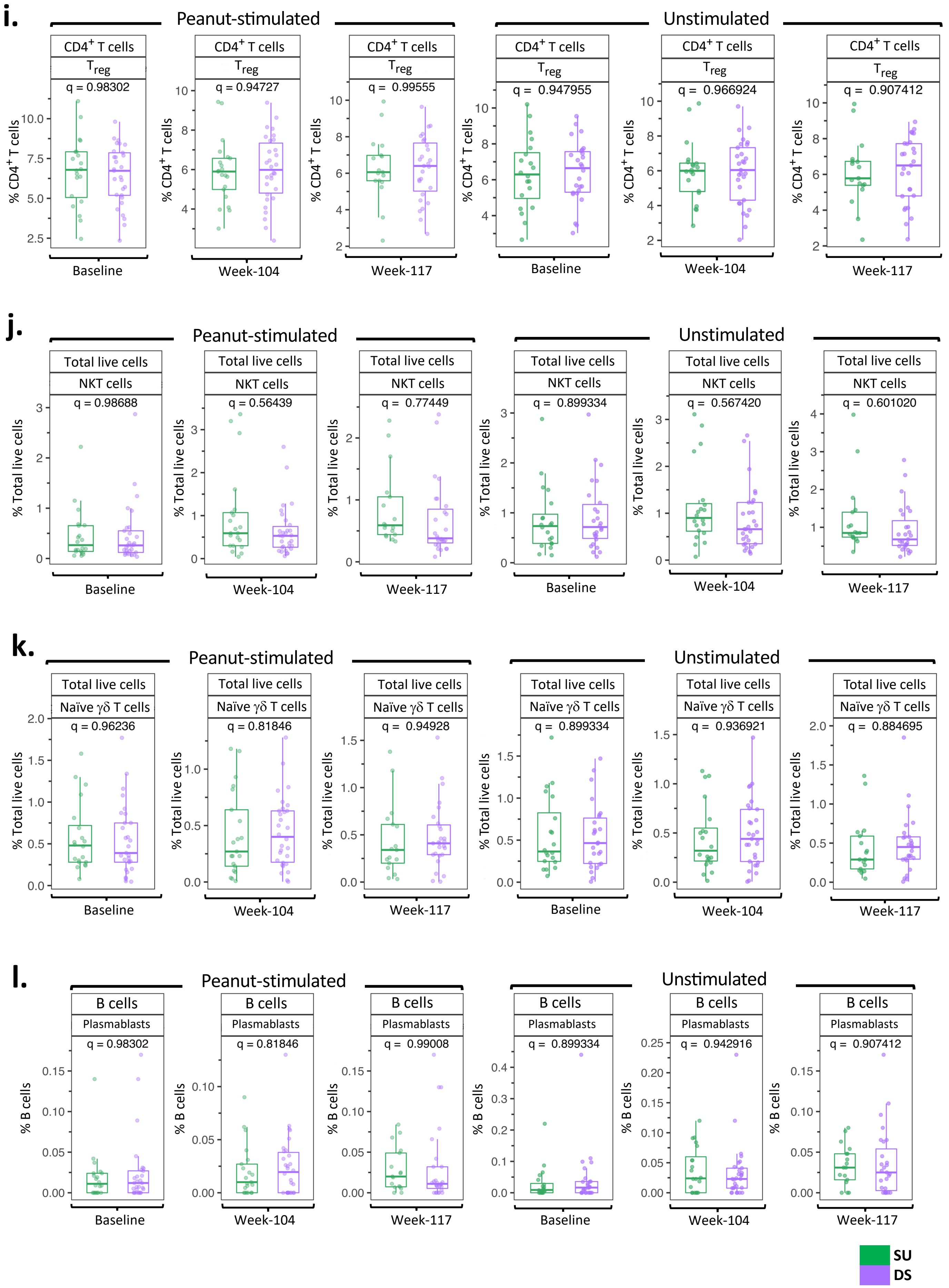
i.
Peanut-stimulated
Unstimulated
CD4+ T cells
Treg
q = 0.98302
q = 0.94727
q = 0.99555
q = 0.947955
q = 0.966924
q = 0.907412
% CD4+ T cells
Baseline
Week-104
Week-117
j.
Peanut-stimulated
Unstimulated
Total live cells
NKT cells
q = 0.98688
q = 0.56439
q = 0.77449
q = 0.899334
q = 0.567420
q = 0.601020
% Total live cells
Baseline
Week-104
Week-117
k.
Peanut-stimulated
Unstimulated
Total live cells
Naïve γδ T cells
q = 0.96236
q = 0.81846
q = 0.94928
q = 0.899334
q = 0.936921
q = 0.884695
% Total live cells
Baseline
Week-104
Week-117
l.
Peanut-stimulated
Unstimulated
B cells
Plasmablasts
q = 0.98302
q = 0.81846
q = 0.99008
q = 0.899334
q = 0.942916
q = 0.907412
% B cells
Baseline
Week-104
Week-117
SU
DS

Supplementary Fig. 3: Frequencies and marker expression of immune cell subsets other than CD8$^+$ T cells remain comparable between SU and DS participants. Immune cell subsets among peanut- stimulated and unstimulated PBMCs from SU participants were compared with DS participants at BL, week 104, and week 117. [Baseline (Peanut stim: SU, n = 20, DS, n = 29; unstim: SU, n = 20, DS, n = 26); Week 104 (Peanut stim: SU, n = 21, DS, n = 30; unstim: SU, n = 20, DS, n = 29); Week 117 (Peanut stim and unstim: SU, n = 17, DS, n = 27)]. Non-significant changes in marker expression and frequency within pr CD4$^+$ and pr CD8$^+$ T cells among peanut-stimulated **(a-h)**, and non-peanut reactive immune cell subsets among peanut- stimulated and unstimulated PBMCs **(i-l)**. In the box-plots, medians are shown, and the ‘hinges’ represent the first and third quartiles. The whiskers are the smallest and largest values after exclusion of outliers (greater than the 75th percentile plus 1.5 times the interquartile range (IQR), or less than 25th percentile minus 1.5 times the IQR. Q values were computed using the χ^2-test in mixed-effects models, adjusted for multiple hypothesis testing using the Benjamini and Hochberg method. Source data are provided as a Source Data file.

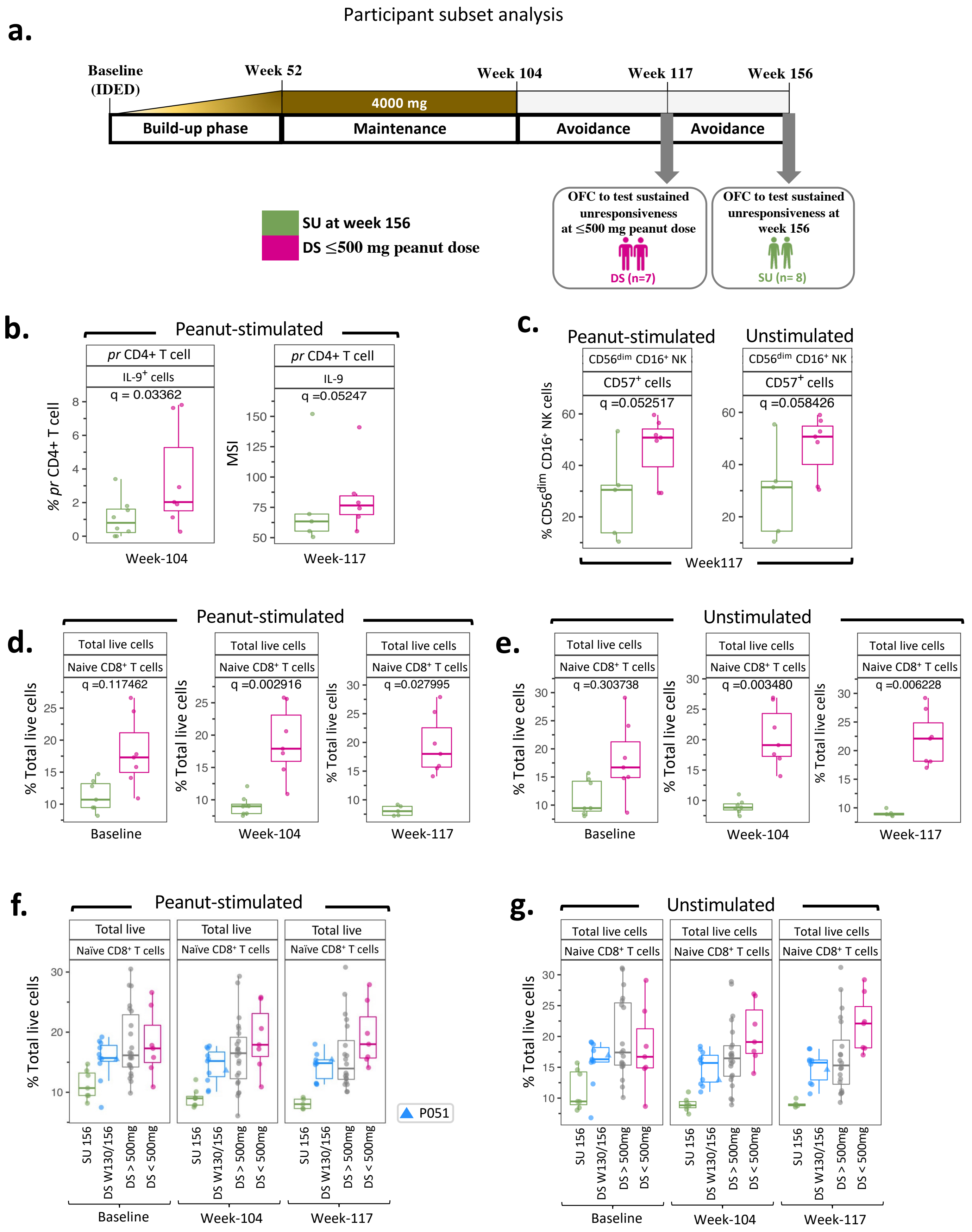
a.
Participant subset analysis
Baseline (IDED)
Week 52
Week 104
Week 117
Week 156
4000 mg
Build-up phase
Maintenance
Avoidance
Avoidance
OFC to test sustained unresponsiveness at ≤500 mg peanut dose
DS (n=7)
OFC to test sustained unresponsiveness at week 156
SU (n= 8)
SU at week 156
DS ≤500 mg peanut dose
b.
Peanut-stimulated
pr CD4+ T cell
IL-9+ cells
q = 0.03362
% pr CD4+ T cell
Week-104
pr CD4+ T cell
IL-9
q =0.05247
MSI
Week-117
c.
Peanut-stimulated
Unstimulated
CD56dim CD16+ NK
CD57+ cells
q =0.052517
q =0.058426
% CD56dim CD16+ NK cells
Week117
d.
Peanut-stimulated
Total live cells
Naive CD8+ T cells
q =0.117462
q =0.002916
q =0.027995
% Total live cells
Baseline
Week-104
Week-117
e.
Unstimulated
q =0.303738
q =0.003480
q =0.006228
f.
Peanut-stimulated
Total live
Naïve CD8+ T cells
SU 156
DS W130/156
DS > 500mg
DS < 500mg
P051
g.
Unstimulated
Total live cells
Naive CD8+ T cells

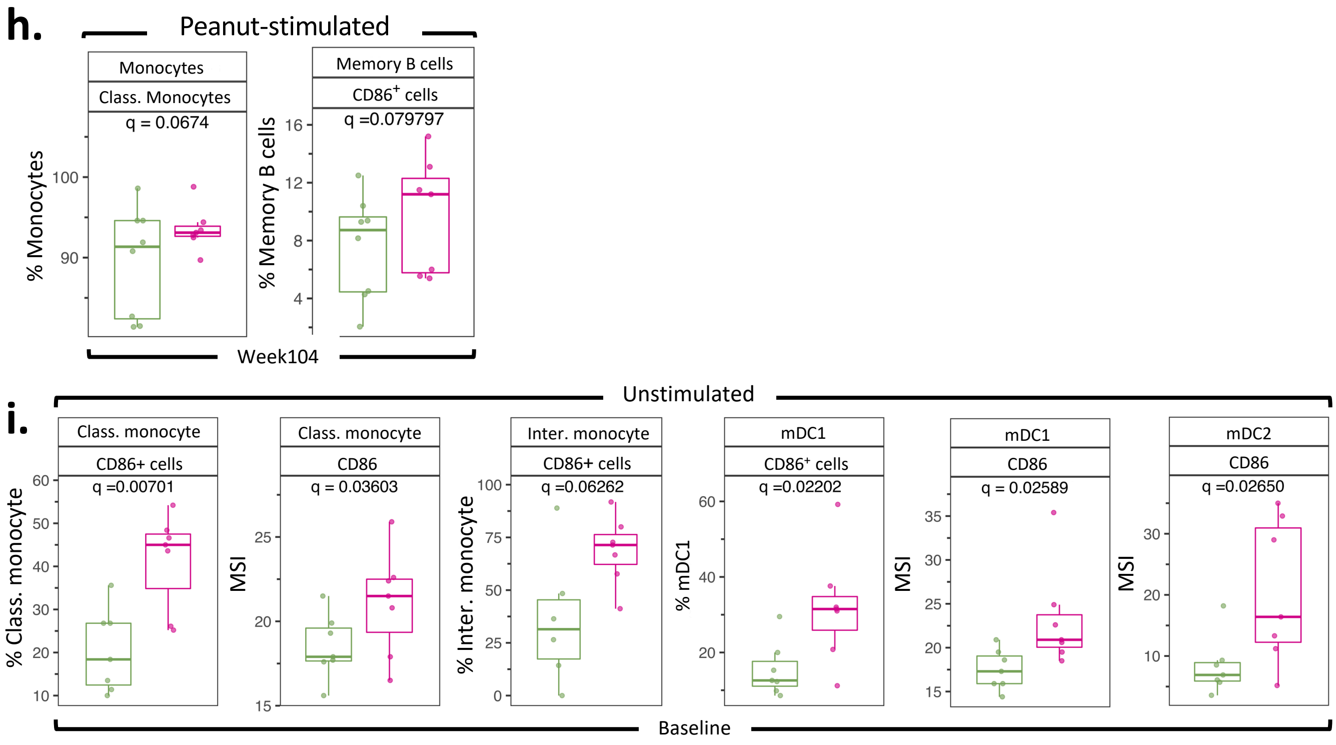

Supplementary Fig. 4: Participants exhibiting long-term SU (SU wk 156) compared with DS ≤ 500 mg showed identical immune distinctions as observed in SU vs. DS comparison. a, Schematic representation of POISED study, showing DBPCFC at week 156 and depiction of subset of SU (SU wk 156) and DS (DS ≤ 500 mg) participants. **b-i**, Frequency and expression changes among immune cell subsets in peanut-stimulated or unstimulated PBMCs at BL, week 104, and week 117 from SU wk 156 participants (n=8) were compared with those from DS ≤ 500 mg participants, who failed a DBPCFC at a dose ≤ 500 mg peanut at week 117 (n=7). Significant differences in frequency or expression of IL-9 in *pr* $CD4^+$ T cells (**b**), CD57 in $CD56^{dim}CD16^+$ NK cells (**c**), naïve $CD8^+$ T cell (**d, e**), and CD86 in APC subsets (**h, i**) are shown. Frequency of naïve $CD8^+$ T cells in peanut-stimulated (**f**) and unstimulated (**g**) PBMCs from all per-protocol avoidance group participants classified by time point and dose of peanut OFC failure. In the box-plots, medians are shown, and the 'hinges' represent the first and third quartiles. The whiskers are the smallest and largest values after exclusion of outliers (greater than the 75th percentile plus 1.5 times the interquartile range (IQR), or less than 25th percentile minus 1.5 times the IQR. Q values were computed using the χ^2-test in mixed-effects models, adjusted for multiple hypothesis testing using the Benjamini and Hochberg method. Source data are provided as a Source Data file.

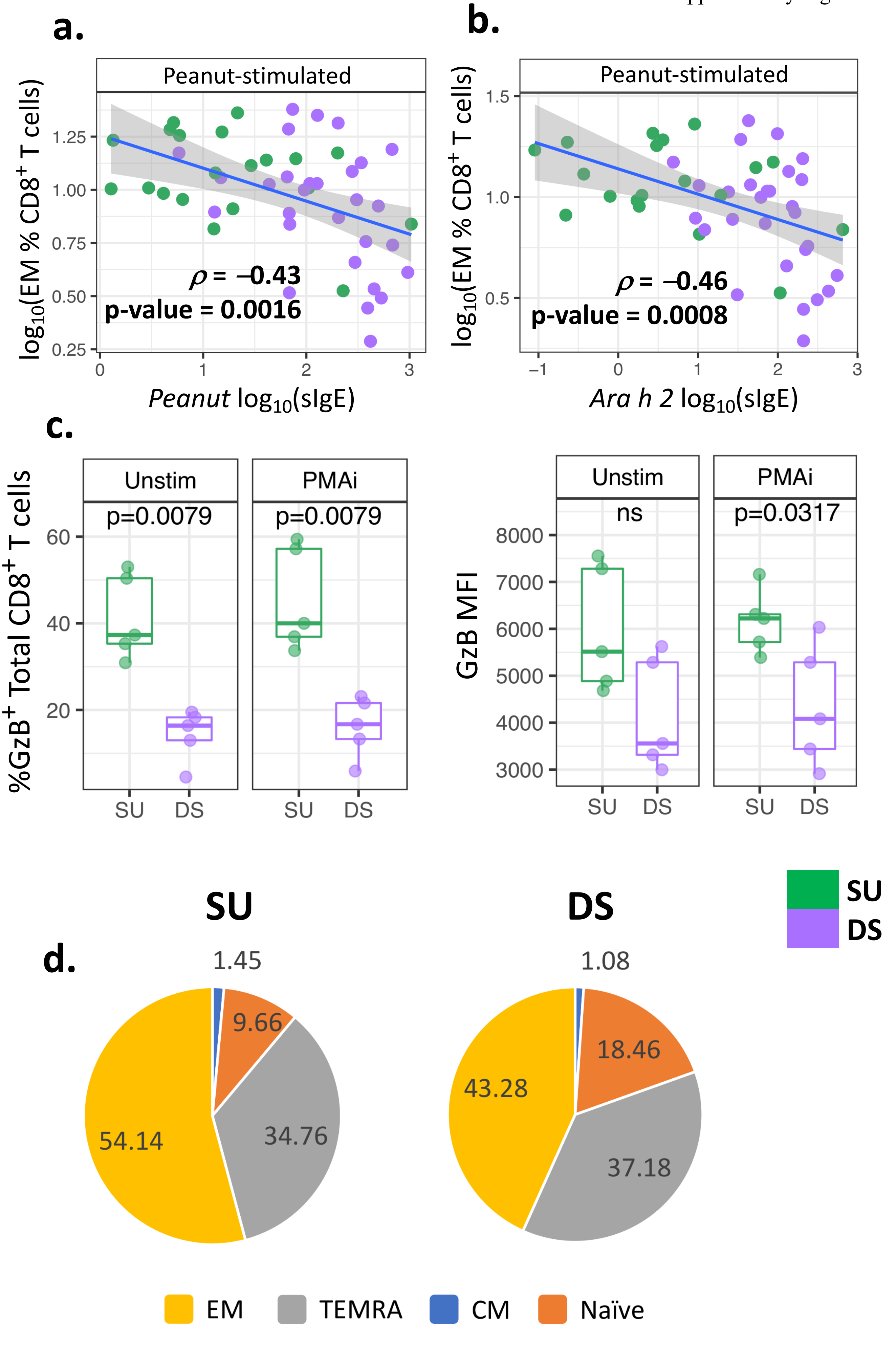
a.
Peanut-stimulated
log10(EM % CD8+ T cells)
1.25
1.00
0.75
0.50
0.25
ρ = –0.43
p-value = 0.0016
0
1
2
3
Peanut log10(sIgE)
b.
Peanut-stimulated
log10(EM % CD8+ T cells)
1.5
1.0
0.5
ρ = –0.46
p-value = 0.0008
–1
0
1
2
3
Ara h 2 log10(sIgE)
c.
%GzB+ Total CD8+ T cells
Unstim
PMAi
p=0.0079
p=0.0079
60
40
20
SU
DS
SU
DS
GzB MFI
Unstim
PMAi
ns
p=0.0317
8000
7000
6000
5000
4000
3000
SU
DS
SU
DS
SU
DS
d.
SU
DS
1.45
9.66
34.76
54.14
1.08
18.46
37.18
43.28
EM
TEMRA
CM
Naïve

Supplementary Fig. 5: Negative correlation of EM CD8$^+$ with peanut- and Ara h 2-specific IgE at BL and distinction in GzB expression among SU vs. DS participants. a,b, Spearman correlations of frequency of EM CD8$^+$ T cells among total CD8$^+$ cells in peanut-stimulated BL PBMCs with peanut-specific (a), and Ara h 2-specific (b) IgE at BL. (pea stim: SU, n = 20, DS, n = 29). Plots display the data points and a linear regression line with a 95% confidence interval shading. P-values in the figure were determined by an unpaired two-tailed Student's t test. c, Frequency (left panels) and expression (right panels) of GzB in total CD8$^+$ T cells among unstimulated and PMAi-stimulated week 52 PBMCs from 5 SU and 5 DS participants evaluated using flow cytometry. **d**, Pie charts depicting relative mean proportion of unstimulated CD8$^+$ T cell subsets expressing GzB in SU vs. DS participants. The whiskers are the smallest and largest values after exclusion of outliers (greater than the 75th percentile plus 1.5 times the interquartile range (IQR), or less than 25th percentile minus 1.5 times the IQR). Plots display the correlation coefficient (ρ) and linear regression line with a 95% confidence interval shading. P value was computed using unpaired, two-sided, non-parametric Mann-Whitney test. Data are presented as the mean ± s.e. Source data are provided as a Source Data file.

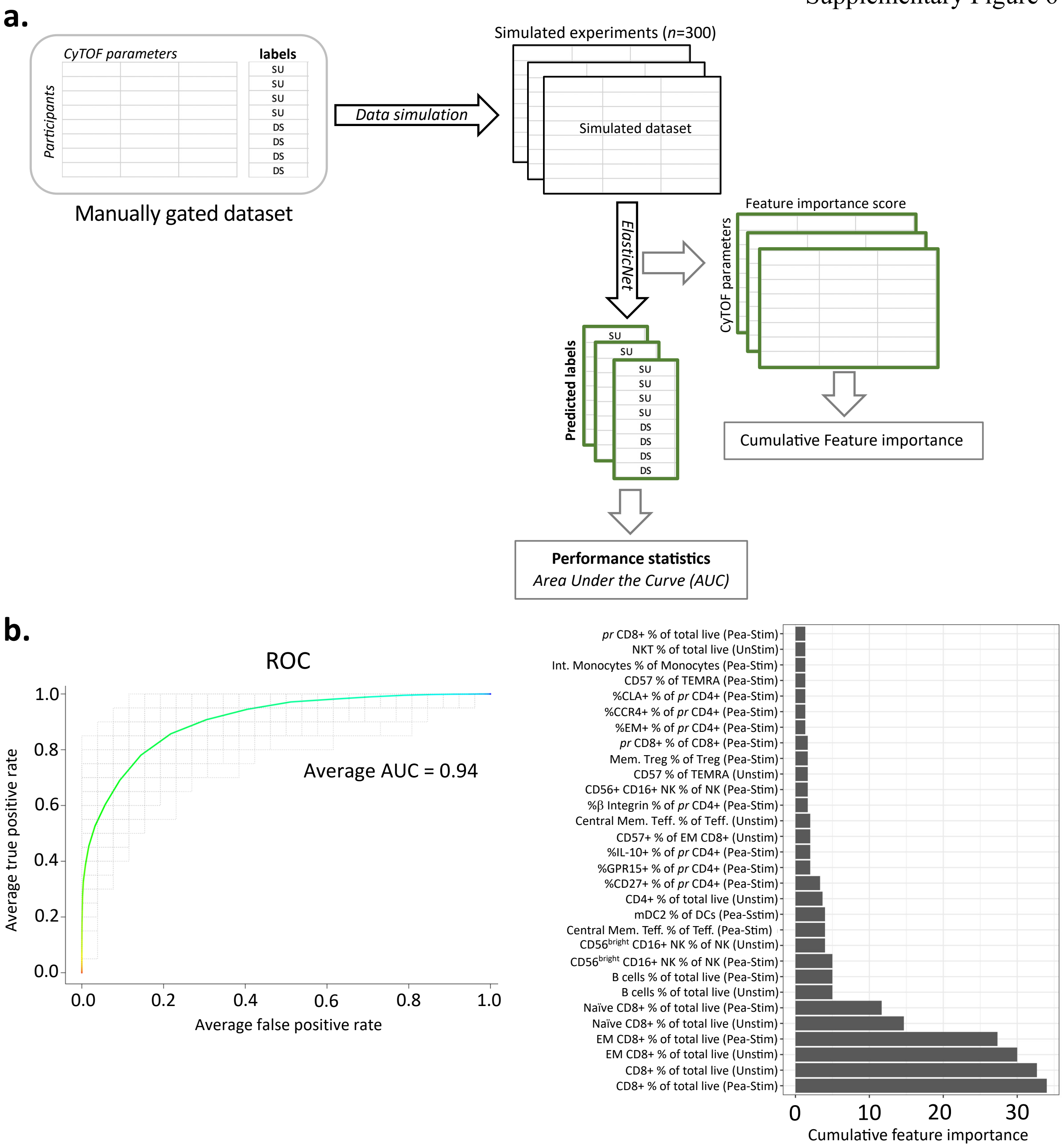

Supplementary Fig. 6: Machine learning analysis underlines CD8^{+} T cell differentiation status as a major predictive feature of SU vs. DS. a, Machine learning analysis workflow. The BL dataset with CyTOF parameters, (i.e., cell type frequencies and marker expression in peanut-simulated and unstimulated PBMCs) was subjected to elastic net modeling for classification of SU vs. DS participants. The performance of elastic net models was evaluated using average AUC and ROC analysis across 300 iterations. The cumulative feature importance score was enumerated for the CyTOF parameters that had feature importance score greater than 80 in at least one elastic net model. **b,** The average AUC and ROC predicted for the given simulated BL datasets. The cumulated feature importance score highlights the most common and important CyTOF parameters used by the models to distinguish SU from DS participants. Source data are provided as a Source Data file.

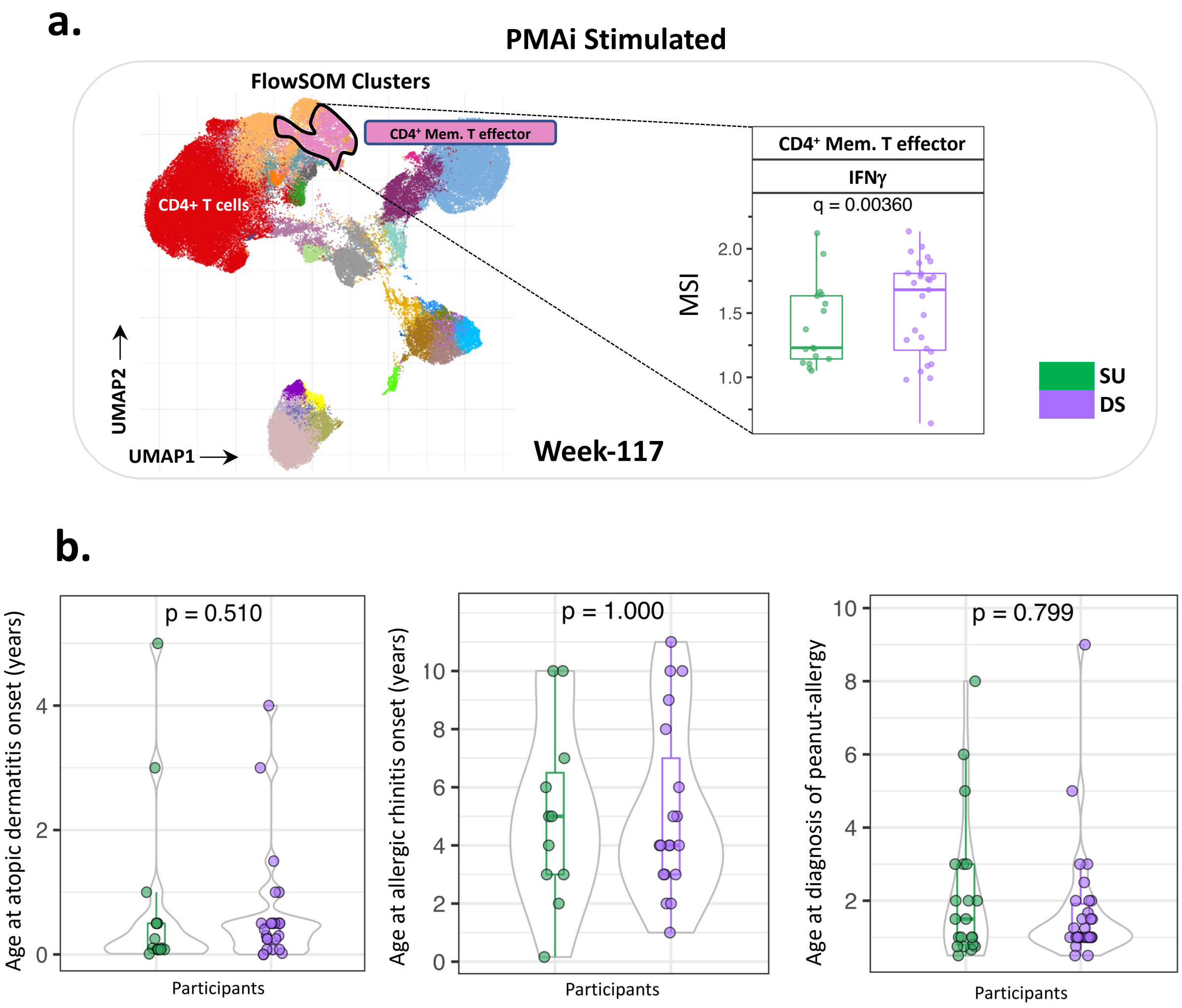

Supplementary Fig. 7: Lower expression of IFNγ in memory CD4$^+$ T effector cluster in SU participants. a, UMAP depicting distinct immune cell subsets identified using FlowSOM-based unsupervised clustering of PMA/Ionomycin-stimulated week 117 PBMCs. Expression of IFNγ in memory CD4$^+$ T effector cluster in SU (n = 17) vs. DS (n = 27) participants. P-values by χ2-tests in mixed-effect model adjusted with Benjamini and Hochberg method. **b,** Comparison of age at onset of atopic dermatitis [SU (n = 15) vs. DS (n = 24)], allergic rhinitis [SU (n = 17) vs. DS (n = 27)], and age at diagnosis of peanut allergy [SU (n = 21) vs. DS (n = 30)]. The whiskers are the smallest and largest values after exclusion of outliers (greater than the 75th percentile plus 1.5 times the interquartile range (IQR), or less than 25th percentile minus 1.5 times the IQR). P value was computed using unpaired, two-sided, non-parametric Mann-Whitney test. Source data are provided as a Source Data file.

a.

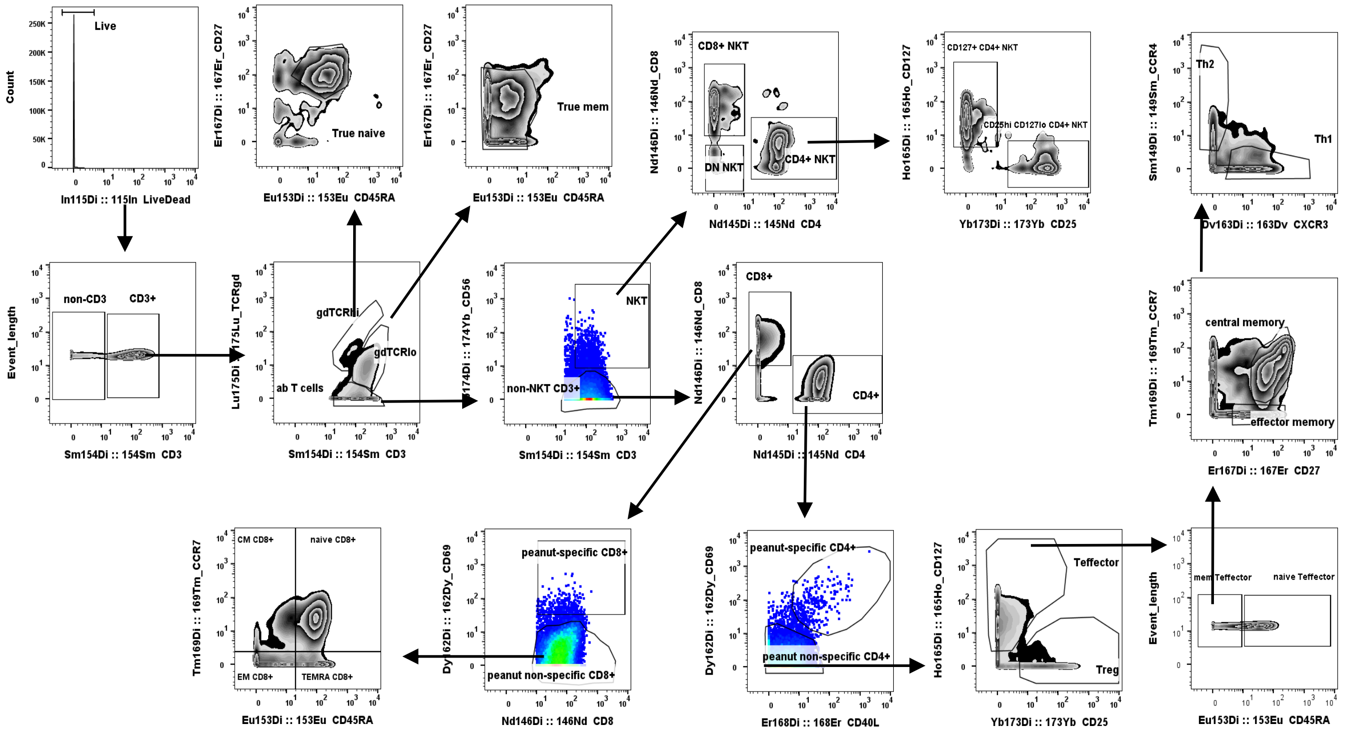
Live
Count
In115Di :: 115In LiveDead
Er167Di :: 167Er_CD27
True naive
Eu153Di :: 153Eu CD45RA
True mem
CD8+ NKT
DN NKT
CD4+ NKT
Nd146Di :: 146Nd_CD8
Nd145Di :: 145Nd CD4
CD127+ CD4+ NKT
CD25hi CD127lo CD4+ NKT
Ho165Di :: 165Ho_CD127
Yb173Di :: 173Yb CD25
Th2
Th1
Sm149Di :: 149Sm_CCR4
Dv163Di :: 163Dv CXCR3
non-CD3
CD3+
Event_length
Sm154Di :: 154Sm CD3
gdTCRhi
gdTCRlo
ab T cells
NKT
non-NKT CD3+
Yb174Di :: 174Yb_CD56
CD8+
CD4+
central memory
effector memory
Tm169Di :: 169Tm_CCR7
Er167Di :: 167Er CD27
CM CD8+
naive CD8+
EM CD8+
TEMRA CD8+
Eu153Di :: 153Eu CD45RA
peanut-specific CD8+
peanut non-specific CD8+
Nd146Di :: 146Nd CD8
peanut-specific CD4+
peanut non-specific CD4+
Dy162Di :: 162Dy_CD69
Er168Di :: 168Er CD40L
Teffector
Treg
Ho165Di :: 165Ho_CD127
Yb173Di :: 173Yb CD25
mem Teffector
naive Teffector

b.

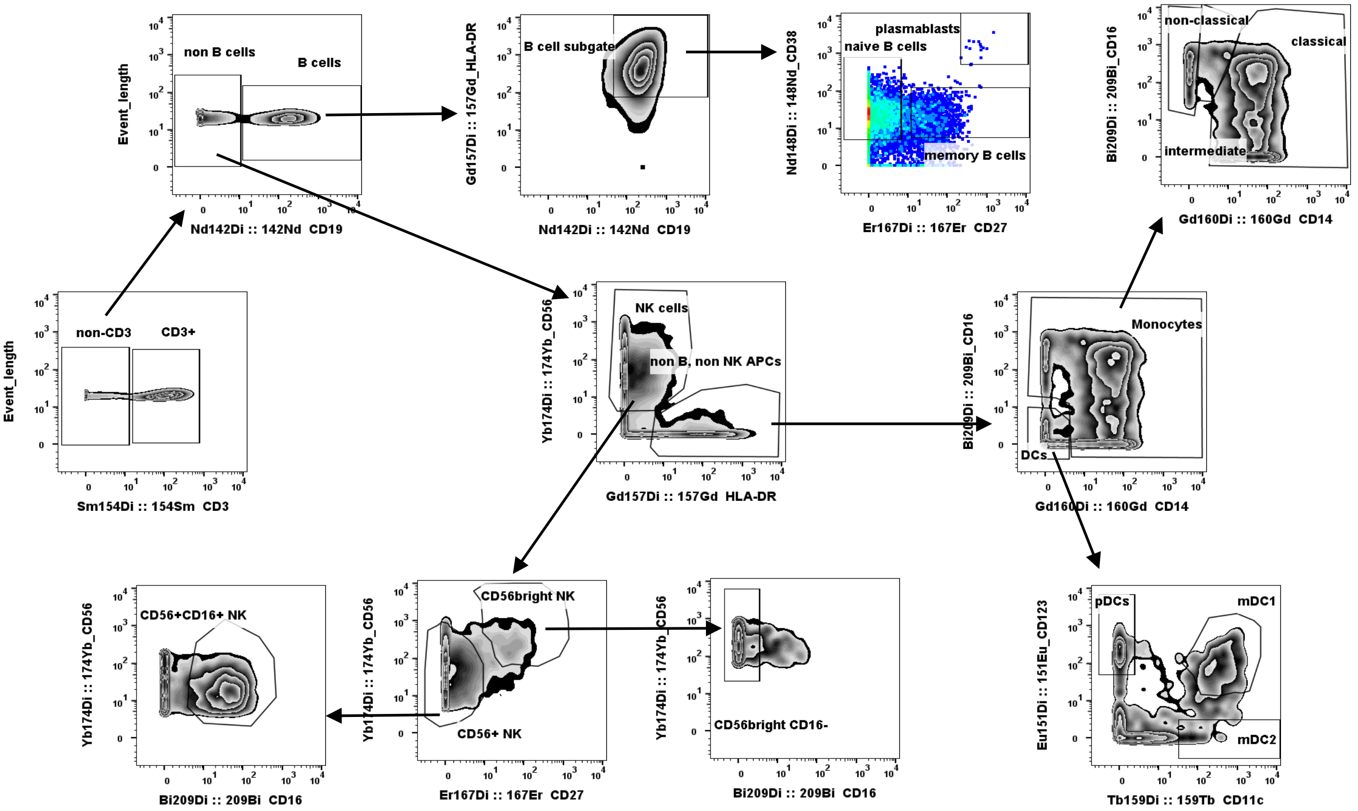
non B cells
B cells
Event_length
Nd142Di :: 142Nd CD19
B cell subgate
Gd157Di :: 157Gd_HLA-DR
plasmablasts
naive B cells
memory B cells
Nd148Di :: 148Nd_CD38
Er167Di :: 167Er CD27
non-classical
classical
intermediate
Bi209Di :: 209Bi_CD16
Gd160Di :: 160Gd CD14
non-CD3
CD3+
Sm154Di :: 154Sm CD3
NK cells
non B, non NK APCs
Yb174Di :: 174Yb_CD56
Gd157Di :: 157Gd HLA-DR
Monocytes
DCs
CD56+CD16+ NK
Bi209Di :: 209Bi CD16
CD56bright NK
CD56+ NK
Er167Di :: 167Er CD27
CD56bright CD16-
pDCs
mDC1
mDC2
Eu151Di :: 151Eu_CD123
Tb159Di :: 159Tb CD11c

Supplementary Fig. 8: **Gating scheme for identification of CD3^{+} and CD3^{-} immune cell subsets among total live PBMCs in CyTOF dataset**. Gating scheme for identification of CD3^{+} and CD3^{-} immune cell subsets among total live PBMCs in CyTOF dataset. PBMCs stimulated with 200 µg/ml peanut solution for 24 hours with the addition of brefeldin A for the last 4 hours were gated using FlowJo v10. **a**, Representative gating for γδ cell subsets, NKT cells, peanut-reactive CD4^{+} T cells, non-peanut reactive CD4^{+} T effector subsets and regulatory T cells, peanut-reactive CD8^{+} T cells, and non peanut-reactive CD8^{+} T cell subsets is depicted. **b**, Representative gating for B cells, NK cells, monocytes, and DC subsets among CD3^{-} parent population is depicted.

Supplementary Table 1. Participant demographics

	CyTOF				
	**Active (n=95)**	**Placebo (n=25)**	**SU (n=21)**	**DS (n=30)**	**Luminex (n=27)**
Age in years, median (IQR, range)	10 (5, 7-47)	11 (5, 7-53)	9 (2, 7-43)	11 (4, 7-29)	9 (4, 7-27)
Female sex, n (%)	24 (30.00%)	6 (26.09%)	7 (33.33%)	9 (30.00%)	8 (29.62%)
Non-Hispanic or Latino ethnicity, n (%)	80 (100.00%)	21 (91.30%)	21 (100.00%)	30 (100.00%)	27 (100%)
History of asthma, n (%)	45 (56.25%)	20 (86.96%)	14 (66.67%)	17 (56.67%)	18 (66.67%)
History of atopic dermatitis, n (%)	56 (70.00%)	16 (69.57%)	15 (71.43%)	24 (80.00%)	19 (70.37%)
History of allergic rhinitis, n (%)	57 (71.25%)	19 (82.61%)	14 (66.67%)	22 (73.33%)	16 (59.25%)
Median CTD of peanut in mg, median (IQR, range)	25 (95, 0-500)	25 (70, 0-375)	75 (150, 5-375)	25 (70, 0-375)	25 (70, 0-375)
Total IgE in kUL/L, median (IQR, range)	552 (977.5, 18-4803)	383 (805.5, 17-4734)	470 (812, 18-2819)	958 (1368.25, 61-4803)	550 (716.5, 35-3225)
Peanut-reactive IgE in kUL/L, median (IQR, range)	76.55 (213.8, 0.62-2186)	74.6 (206.34, 0.41-964)	13 (26.22, 0.76-2186)	139 (360.4, 8.57-854)	89.5 (401.95, 0.76-2186)
Median SPT wheel diameter of peanut in mm, median (IQR, range)	12 (7.625, 4-29.5)	12 (5, 6-33)	12 (10, 4-23.5)	12.25 (7.375, 5-29.5)	11 (6.5, 4-22)

Supplementary Table 2. Sample count

	CyTOF			Luminex		
	PBMC			Supernatant		Plasma
Time / Stimulation	**Peanut stim**	**Unstim**	**PMAi stim**	**Peanut stim**	**Unstim**	-
**Baseline**<br>[#SU; #DS]	110<br>[20; 29]	107<br>[20; 26]	109<br>[20; 27]	19<br>[07;07]	19<br>[07;07]	24<br>[08;10]
**Week-104**<br>[#Active; #Placebo]<br>[#SU; #DS]	95<br>[80; 15]<br>[21;30]	91<br>[76; 15]<br>[20;29]	93<br>[78; 15]<br>[21;29]	24<br>[16; 07]<br>[10;07]	23<br>[16; 07]<br>[07;09]	28<br>[21; 07]<br>[08;13]
**Week-117**<br>[#SU; #DS]	75<br>[17;27]	72<br>[17;27]	74<br>[17;27]	17<br>[07;09]	16<br>[07;09]	21<br>[08;13]

Supplementary Table 3. Lineage and functional markers used in FlowSOM unsupervised clustering.

Stimulation	Lineage Markers	Functional markers
Peanut-stimulated and Unstimulated samples	CD19, CD49b, CD4, CD8, CD20, CD38, CCR4, LAG3, CD123, CD45RA, CD3, HLA-DR, CD33, CD11c, CD14, CD69, CXCR3, CD27, CD40L, CCR7, CD25, CD56, TCRγδ, CD16	CD86, OX40, CD28, GPR15, PD1, integrin, CLA, CD57, IL-4, IL-9, IFNγ, IL-17, LAP, IL-10
PMA/ionomycin-stimulated samples	CD19, CD4, CD8, CD20, CD38, CD45RA, CD3, HLA-DR, CD11c, CD14, CD27, CCR7, CD25, CD56, TCRγδ	IL-4, IL-9, IFNγ, IL-17, LAP, IL-10
