# Supplementary material for: CD8+ T cell differentiation status correlates with the feasibility of sustained unresponsiveness following oral immunotherapy
Source: Nat Commun. 2022 Nov 4;13:6646. doi: 10.1038/s41467-022-34222-8 (PMC9636180; doi:10.1038/s41467-022-34222-8)
Supplement: Supplementary file 2 — Description of Additional Supplementary Files [file 41467_2022_34222_MOESM2_ESM.pdf]

Description of Additional Supplementary Files

File Name: Supplementary Data 1

Description: [Tab-1]: **List of 40 metal-conjugated antibodies** used for mass cytometry (CyTOF) along with their respective catalog number, vendor, and other relevant information. [Tab-2]: **List of 7 fluorescent antibodies** used to evaluate Granzyme B output from $CD8^{+}$ T cell subset by flow cytometry. Vendor, catalog numbers and other relevant information are listed along with each antibody.
