# Supplementary material for: CD8+ T cell differentiation status correlates with the feasibility of sustained unresponsiveness following oral immunotherapy
Source: Nat Commun. 2022 Nov 4;13:6646. doi: 10.1038/s41467-022-34222-8 (PMC9636180; doi:10.1038/s41467-022-34222-8)
Supplement: Supplementary file 4 — Reporting Summary [file 41467_2022_34222_MOESM4_ESM.pdf]

nature research

Corresponding author(s): Kari C Nadeau

Last updated by author(s): Sep 26, 2022

Reporting Summary

Nature Research wishes to improve the reproducibility of the work that we publish. This form provides structure for consistency and transparency in reporting. For further information on Nature Research policies, see our Editorial Policies and the Editorial Policy Checklist.

Statistics

For all statistical analyses, confirm that the following items are present in the figure legend, table legend, main text, or Methods section.

n/a	Confirmed	
☐	☒	The exact sample size (*n*) for each experimental group/condition, given as a discrete number and unit of measurement
☐	☒	A statement on whether measurements were taken from distinct samples or whether the same sample was measured repeatedly
☐	☒	The statistical test(s) used AND whether they are one- or two-sided<br>*Only common tests should be described solely by name; describe more complex techniques in the Methods section.*
☐	☒	A description of all covariates tested
☒	☐	A description of any assumptions or corrections, such as tests of normality and adjustment for multiple comparisons
☐	☒	A full description of the statistical parameters including central tendency (e.g. means) or other basic estimates (e.g. regression coefficient) AND variation (e.g. standard deviation) or associated estimates of uncertainty (e.g. confidence intervals)
☐	☒	For null hypothesis testing, the test statistic (e.g. *F*, *t*, *r*) with confidence intervals, effect sizes, degrees of freedom and *P* value noted<br>*Give P values as exact values whenever suitable.*
☒	☐	For Bayesian analysis, information on the choice of priors and Markov chain Monte Carlo settings
☒	☐	For hierarchical and complex designs, identification of the appropriate level for tests and full reporting of outcomes
☐	☒	Estimates of effect sizes (e.g. Cohen's *d*, Pearson's *r*), indicating how they were calculated

Our web collection on statistics for biologists contains articles on many of the points above.

Software and code

Policy information about availability of computer code

Data collection	No Software was used to collect any publicly or commercialy available dataset.
Data analysis	R (version 3.6): The R Foundation for Statistical Computing Platform.<br>R package FlowSOM (Version 2.2.0): Using self-organizing maps for visualization and interpretation of cytometry data<br>R package UMAP (version 0.2.7.0): Uniform Manifold Approximation and Projection<br>R package ggplot (version 3.3.3): Create Elegant Data Visualisations Using the Grammar<br>R package elasticnet (version 1.3): Elastic-Net for Sparse Estimation and Sparse PCA<br>R pckage lmerTest (Version 3.1-3): Tests in Linear Mixed Effects Models<br>R package pheatmap (version 1.0.12): A function to draw clustered heatmaps.<br>R Package flowStats (version 4.4.0)<br>R package rstatix (Version 0.7.0)<br>Helios Mass Cytometer Software v7.0 (Fluidigm, South San Francisco, CA, USA)<br>FlowJo (V10)<br>BioRender online application

For manuscripts utilizing custom algorithms or software that are central to the research but not yet described in published literature, software must be made available to editors and reviewers. We strongly encourage code deposition in a community repository (e.g. GitHub). See the Nature Research guidelines for submitting code & software for further information.

Data

Policy information about availability of data

All manuscripts must include a data availability statement. This statement should provide the following information, where applicable:

- Accession codes, unique identifiers, or web links for publicly available datasets
- A list of figures that have associated raw data
- A description of any restrictions on data availability

Raw unlabeled POISED Mass Cytometry dataset files in FCS 3.0 format and associated meta-data as well as gating schema can be obtained from FlowRepository (FR-FCM-Z4MA and FR-FCM-Z2V9). Wherein, experiment ID FR-FCM-Z4MA may be accessed via the following URL:
https://flowrepository.org/id/RvFrOki98cB1R0srOsojhr4rTJpjCchGopou7h7BCE2CAS6fYgNWUdFLnbCSEO38.
Please share this URL with the reviewers.

Field-specific reporting

Please select the one below that is the best fit for your research. If you are not sure, read the appropriate sections before making your selection.

☒ Life sciences ☐ Behavioural & social sciences ☐ Ecological, evolutionary & environmental sciences

For a reference copy of the document with all sections, see nature.com/documents/nr-reporting-summary-flat.pdf

Life sciences study design

All studies must disclose on these points even when the disclosure is negative.

Sample size	The sample size calculation was performed and reported in the POISED clinical trial original publication (PMID: 31522849). In the current post-hoc mechanistic analysis, we applied stringent statistical tests corrected for batch effects along with multiple hypothesis testing.
Data exclusions	No data were excluded from this study
Replication	Mass cytomtery analysis was performed as a single experiment per sample. Luminex analysis was perfomed a single experiment per sample with technical replicates (duplicates).
Randomization	Randomization details have been reported in the POISED clinical trial original publication (PMID: 31522849).
Blinding	Investigators were blinded during the original clinical trial (PMID: 31522849) but not in the current post-hoc mechanistic analysis.

Reporting for specific materials, systems and methods

We require information from authors about some types of materials, experimental systems and methods used in many studies. Here, indicate whether each material, system or method listed is relevant to your study. If you are not sure if a list item applies to your research, read the appropriate section before selecting a response.

Materials & experimental systems

n/a	Involved in the study
☐	☒ Antibodies
☒	☐ Eukaryotic cell lines
☒	☐ Palaeontology and archaeology
☒	☐ Animals and other organisms
☐	☒ Human research participants
☒	☐ Clinical data
☒	☐ Dual use research of concern

Methods

n/a	Involved in the study
☒	☐ ChIP-seq
☐	☒ Flow cytometry
☒	☐ MRI-based neuroimaging

Antibodies

Antibodies used

Metal label,Specificity,Clone ,Catalogue #,Source,Metal conjugation,Dilution,Surfcae vs. Intracellular
89Y,CD57,HCD57,322302,Biolegend ,in-house,1:50,Surface
115In,live/dead,maleimide-monoamide-DOTA,B-272,Macrocyclics,commercially available,2.125,Surface
113In,CD86,IT2.2,305435,Biolegend,in-house,1:50,Surface
141Pr,OX40,ACT35,555836,BD,in-house,1:50,Surface
142Nd,CD19,HIB19,3142001B,Fluidigm,commercially available,1:100,Surface
143Nd,CD49b,P1E6-C5,359301,Biolegend,in-house,1:50,Surface
144Nd,IL-4,MP4-25D2,3144010B,Fluidigm,commercially available,1:100,Intracellular

145Nd,CD4,RPA-T4,3145001B,Fluidigm,commercially available,1:100,Surface
146Nd,CD8,RPA-T8,3146001B,Fluidigm,commercially available,1:100,Surface
147Sm,CD20,2H7,3147001B,Fluidigm,commercially available,1:100,Surface
148Nd,CD38,HB-7,356602,Biolegend,in-house,1:200,Surface
149Sm,CCR4,205410,3149003A,Fluidigm,commercially available,1:100,Surface
150Nd,LAG3,874501,3150016B,Fluidigm,commercially available,1:100,Surface
151Eu,CD123,6H6,3151001B,Fluidigm,commercially available,1:100,Surface
152Sm,IL9,MH9A3,507704,Biolegend,in-house,1:50,Intracellular
153Eu,CD45RA,HI100,3153001B,Fluidigm,commercially available,1:100,Surface
154Sm,CD3,UCHT1,3154003B,Fluidigm,commercially available,1:200,Surface
155Gd,CD28,L283,340975,BD,commercially available,1:200,Surface
156Gd,GPR15,SA302A10,custom,Biolegend,in-house,1:50,Surface
157Gd,HLA-DR,G46-6,555810,BD,in-house,1:200,Surface
158Gd,CD33,WM53,3158001B,Fluidigm,commercially available,1:100,Surface
159Tb,CD11c,Bu15,3159001,Fluidigm,commercially available,1:200,Surface
160Gd,CD14,M5E2,3160001B,Fluidigm,commercially available,1:100,Surface
161Dy,IFNg,4S.B4, 147319-85,ebioscience,in-house,1:200,Intracellular
162Dy,CD69,FN50,3162001B,Fluidigm,commercially available,1:100,Intracellular
163Dy,CXCR3,G025H7,3163004B,Fluidigm,commercially available,1:100,Surface
164Dy,IL-17,N49-853,3164002B,Fluidigm,commercially available,1:100,Intracellular
165Ho,CD127,A019D5,3165008B,Fluidigm,commercially available,1:100,Surface
166Er,LAP,TW7-28G11,146703,Biolegend,in-house,1:50,Intracellular
167Er,CD27,L128,3167006B,Fluidigm,commercially available,1:100,Surface
168Er,CD40L,24-31,3168006B,Fluidigm,commercially available,1:100,Intracellular
169Tm,CCR7,G043H7,35322,Biolegend,in-house,1:200,Surface
170Er,PD1,EH12.1,562138,BD,in-house,1:100,Surface
171Yb,IL-10,JES3-9D7,501402,Biolegend,in-house,1:50,Intracellular
172Yb,beta 7 integrin,FIB27,121003,Biolegend,in-house,1:100,Surface
173Yb,CD25,M-A251,555430,BD,in-house,1:100,Surface
174Yb,CD56,NCAM16.2,559043,BD,in-house,1:50,Surface
175Lu,TCRgd,B1,331202,Biolegend,in-house,1:50,Surface
176Yb,CLA,HECA452,3176018B,Fluidigm,commercially available,1:100,Surface
209Bi,CD16,3G8,3209002B,Fluidigm,commercially available,1:100,Surface

Validation

For in-house metal conjugation, purified antibodies were purchased from BD Biosciences or Biolegend. Each in-house conjugated antibody per batch of conjugation was tested and titrated using unstimulated and PMA/Ionomycin-stimulated PBMCs isolated from a buffy coat sample. Metal pre-conjugated, QA/QCed, ready-to-use antibodies were purchased from Fluidigm. The whole antibody panel was tested and validated for it's function using unstimulated and PMA/Ionomycin-stimulated PBMCs isolated from a buffy coat sample.

Human research participants

Policy information about studies involving human research participants

Population characteristics

participant_id,Gender,age_of_enrollment,Age_diagnosis_pea_allergy,History of asthma?,History of allergic rhinitis?,History of atopic dermatitis?,Ethnicity:
P001,Male,9,2,Yes,No,Yes,Not Hispanic or Latino
P002,Male,8,1,No,Yes,Yes,Not Hispanic or Latino
P003,Male,8,3,Yes,Yes,Yes,Not Hispanic or Latino
P004,Female,9,1.5,Yes,Yes,Yes,Not Hispanic or Latino
P005,Male,11,0.75,Yes,Yes,Yes,Not Hispanic or Latino
P006,Male,12,1,Yes,Yes,Yes,Not Hispanic or Latino
P007,Male,10,1,Yes,Yes,Yes,Not Hispanic or Latino
P008,Male,16,1,Yes,Yes,Yes,Not Hispanic or Latino
P009,Male,8,1.16,No,Yes,Yes,Not Hispanic or Latino
P010,Female,10,1.67,Yes,Yes,Yes,Not Hispanic or Latino
P011,Male,11,1,No,Yes,Yes,Not Hispanic or Latino
P012,Male,9,1,Yes,No,Yes,Not Hispanic or Latino
P013,Male,13,1,No,Yes,Yes,Not Hispanic or Latino
P014,Female,9,1,Yes,Yes,Yes,Not Hispanic or Latino
P015,Female,12,6,Yes,Yes,No,Not Hispanic or Latino
P016,Male,9,2,Yes,Yes,Yes,Not Hispanic or Latino
P017,Male,10,0.92,No,Yes,Yes,Not Hispanic or Latino
P018,Male,12,1,Yes,Yes,Yes,Not Hispanic or Latino
P019,Male,9,2,Yes,Yes,Yes,Not Hispanic or Latino
P020,Male,8,2,Yes,Yes,Yes,Not Hispanic or Latino
P021,Male,11,1,Yes,Yes,Yes,Not Hispanic or Latino
P022,Male,11,1.25,No,Yes,Yes,Not Hispanic or Latino
P023,Female,8,3,Yes,Yes,Yes,Not Hispanic or Latino
P024,Male,9,0.75,Yes,Yes,Yes,Not Hispanic or Latino
P025,Male,10,2,Yes,Yes,Yes,Not Hispanic or Latino
P026,Male,9,1.7,Yes,Yes,No,Hispanic or Latino
P027,Female,12,1.25,No,Yes,Yes,Not Hispanic or Latino
P028,Male,11,1,No,Yes,Yes,Not Hispanic or Latino
P029,Female,15,1,No,No,No,Not Hispanic or Latino
P030,Female,17,2,No,No,No,Not Hispanic or Latino
P031,Male,9,3,No,No,No,Not Hispanic or Latino
P032,Male,9,0.66,No,Yes,No,Not Hispanic or Latino
P033,Female,10,0.8,No,No,Yes,Not Hispanic or Latino
P034,Female,14,0.9,No,Yes,Yes,Not Hispanic or Latino
P035,Male,8,0.8,Yes,Yes,Yes,Not Hispanic or Latino
P036,Male,14,0.75,Yes,Yes,Yes,Not Hispanic or Latino
P037,Male,10,1,Yes,Yes,Yes,Not Hispanic or Latino
P038,Male,11,0.5,Yes,Yes,Yes,Not Hispanic or Latino
P039,Male,9,2,Yes,No,Yes,Not Hispanic or Latino
P040,Female,8,0.5,Yes,Yes,Yes,Not Hispanic or Latino
P041,Female,8,1.5,Yes,Yes,No,Not Hispanic or Latino
P042,Male,9,1.5,Yes,Yes,Yes,Not Hispanic or Latino
P043,Male,9,1,No,No,Yes,Not Hispanic or Latino
P044,Male,12,1.5,No,Yes,No,Not Hispanic or Latino
P045,Male,12,3,Yes,Yes,No,Not Hispanic or Latino
P046,Male,11,1.2,No,Yes,Yes,Not Hispanic or Latino
P047,Male,15,1.25,No,No,Yes,Not Hispanic or Latino
P048,Male,13,3,No,Yes,Yes,Not Hispanic or Latino
P049,Male,12,1,No,Yes,Yes,Not Hispanic or Latino
P050,Female,11,2,Yes,No,Yes,Not Hispanic or Latino
P051,Female,9,1,No,No,No,Not Hispanic or Latino
P052,Female,16,2,Yes,No,Yes,Not Hispanic or Latino
P053,Female,18,1,Yes,No,Yes,Not Hispanic or Latino
P054,Female,11,1,Yes,Yes,Yes,Not Hispanic or Latino
P055,Male,12,1.5,Yes,Yes,Yes,Not Hispanic or Latino
P056,Female,14,11,No,No,No,Not Hispanic or Latino
P057,Male,10,0.75,Yes,No,Yes,Not Hispanic or Latino
P058,Male,9,0.8,Yes,Yes,Yes,Not Hispanic or Latino
P059,Male,8,2.5,No,No,Yes,Not Hispanic or Latino
P060,Female,15,3,Yes,Yes,Yes,Hispanic or Latino
P061,Male,10,0.5,Yes,Yes,Yes,Not Hispanic or Latino
P062,Female,13,2,No,Yes,Yes,Not Hispanic or Latino

P063,Female,13,1.5,Yes,Yes,No,Not Hispanic or Latino
P064,Male,9,3,No,Yes,Yes,Not Hispanic or Latino
P065,Male,8,2,Yes,No,No,Not Hispanic or Latino
P066,Male,9,1,Yes,Yes,Yes,Not Hispanic or Latino
P067,Male,8,3,Yes,Yes,Yes,Not Hispanic or Latino
P068,Male,9,2,Yes,Yes,Yes,Not Hispanic or Latino
P069,Male,8,5,Yes,No,No,Not Hispanic or Latino
P070,Male,9,3,Yes,Yes,Yes,Not Hispanic or Latino
P071,Female,12,0.67,Yes,Yes,Yes,Not Hispanic or Latino
P072,Female,8,1,No,No,No,Not Hispanic or Latino
P073,Female,8,1,Yes,No,Yes,Not Hispanic or Latino
P074,Male,8,0.67,No,Yes,Yes,Not Hispanic or Latino
P075,Male,16,3,Yes,Yes,Yes,Not Hispanic or Latino
P076,Male,13,1,No,Yes,Yes,Not Hispanic or Latino
P077,Female,8,1,Yes,No,Yes,Not Hispanic or Latino
P078,Male,12,4,Yes,Yes,No,Not Hispanic or Latino
P079,Female,7,1,Yes,Yes,Yes,Not Hispanic or Latino
P080,Female,8,0.75,Yes,No,No,Not Hispanic or Latino
P081,Female,7,2,No,Yes,No,Not Hispanic or Latino
P082,Male,14,1.5,Yes,Yes,Yes,Not Hispanic or Latino
P083,Female,7,1.5,No,No,Yes,Not Hispanic or Latino
P084,Male,7,1,Yes,No,Yes,Not Hispanic or Latino
P085,Male,16,0.5,Yes,Yes,Yes,Hispanic or Latino
P086,Male,11,3,Yes,Yes,Yes,Not Hispanic or Latino
P087,Female,7,1.2,Yes,No,Yes,Not Hispanic or Latino
P088,Female,8,5,No,Yes,Yes,Not Hispanic or Latino
P089,Male,7,3,Yes,Yes,No,Not Hispanic or Latino
P090,Male,7,2,Yes,Yes,Yes,Not Hispanic or Latino
P091,Female,13,2.5,Yes,Yes,No,Not Hispanic or Latino
P092,Female,7,3,Yes,Yes,Yes,Not Hispanic or Latino
P093,Male,8,2.5,No,No,No,Not Hispanic or Latino
P094,Male,7,2,Yes,Yes,Yes,Not Hispanic or Latino
P095,Male,7,2,Yes,Yes,Yes,Not Hispanic or Latino
P096,Female,7,1,No,Yes,Yes,Not Hispanic or Latino
P097,Male,7,1.5,No,Yes,Yes,Not Hispanic or Latino
P098,Male,7,1.5,No,Yes,No,Not Hispanic or Latino
P099,Male,16,1,No,No,No,Not Hispanic or Latino
P100,Male,47,5,Yes,Yes,No,Not Hispanic or Latino
P101,Male,29,2,Yes,Yes,No,Not Hispanic or Latino
P102,Male,33,3,No,Yes,No,Not Hispanic or Latino
P103,Male,22,8,Yes,Yes,No,Not Hispanic or Latino
P104,Male,27,2,Yes,No,No,Not Hispanic or Latino
P105,Male,35,8,Yes,Yes,No,Not Hispanic or Latino
P106,Male,53,2,Yes,Yes,No,Not Hispanic or Latino
P107,Female,26,2,No,No,Yes,Not Hispanic or Latino
P108,Male,22,3,Yes,Yes,No,Not Hispanic or Latino
P109,Female,47,0.33,Yes,Yes,Yes,Not Hispanic or Latino
P110,Female,29,1,Yes,Yes,Yes,Not Hispanic or Latino
P111,Male,20,4,Yes,No,Yes,Not Hispanic or Latino
P112,Male,49,0.5,Yes,Yes,Yes,Not Hispanic or Latino
P113,Male,43,6,Yes,Yes,No,Not Hispanic or Latino
P114,Male,19,1.5,No,Yes,Yes,Not Hispanic or Latino
P115,Male,24,5,Yes,Yes,Yes,Not Hispanic or Latino
P116,Male,22,1,Yes,Yes,Yes,Not Hispanic or Latino
P117,Female,24,3,No,Yes,Yes,Not Hispanic or Latino
P118,Male,31,3,Yes,Yes,No,Not Hispanic or Latino
P119,Male,26,9,Yes,Yes,Yes,Not Hispanic or Latino
P120,Female,26,0.5,Yes,Yes,Yes,Not Hispanic or Latino

Recruitment

Criteria for patient recruitment is available in the original research article. Please refer to DOI: https://doi.org/10.1016/S0140-6736(19)31793-3

Ethics oversight

The clinical research protocol for POISED study was approved by the Division of Allergy, Immunology, and Transplantation (DAIT) and the National Institute of Allergy and Infectious Diseases (NIAID) Allergy and Asthma Data Safety Management Board, the DAIT/NIAID Clinical Review Committee, the Stanford Institutional Review Board, and the US Food and Drug Administration (FDA). Written informed consent was obtained from adult participants or parents/ guardians of minor participants along with assent from minor participants of age 7 years and older. All compliant participants were given $30 gift cards as a compensation for each study visit as consistent with Stanford IRB-approved protocol.

Note that full information on the approval of the study protocol must also be provided in the manuscript.

Flow Cytometry

Plots

Confirm that:

- [x] The axis labels state the marker and fluorochrome used (e.g. CD4-FITC).
- [x] The axis scales are clearly visible. Include numbers along axes only for bottom left plot of group (a 'group' is an analysis of identical markers).
- [ ] All plots are contour plots with outliers or pseudocolor plots.
- [x] A numerical value for number of cells or percentage (with statistics) is provided.

Methodology

Sample preparation	From each study participant, 40 mL blood was drawn by venipuncture at baseline of a peanut oral immunotherapy trials. Using validated, standardized, and published procedures, PBMCs and plasma isolated by ficoll-based density gradient centrifugation were frozen in aliquots, and stored in liquid nitrogen and at -80°C, respectively, until thawing for respective assays. In this analysis, we used samples taken at baseline. In-house metal conjugation of purified antibodies as indicated in table S1 was carried out using Maxpar antibody labeling kits (Fluidigm Co., South San Francisco, CA). PBMCs were thawed and rested overnight at 37°C with 5% CO2 in a combination of RPMI, 10% FBS, and Pen-Strep. Cells were plated in a round-bottom, 96-well plate at the density of 3e6 cells in culture with 300 uL RPMI, 5% FBS, Pen-Strep per well. To evaluate peanut-specific response, PBMCs were stimulated with 200 ug/mL peanut solution for 24 hours with the addition of brefeldin A (5 ug/mL; Biolegend, San Diego, CA) for the last 4 hours. Unstimulated cells treated with brefeldin A for 4 hours before harvesting served as controls. Cells were harvested and stained as previously published with an added barcoding step post-permeabilizing the cells. Cell-ID 20-plex Palladium barcoding set (Fluidigm Co.) was used for barcoding according to manufacturer's instructions. Barcoded and pooled samples were acquired on a Helios mass cytometer (Fluidigm Co.).
Instrument	Helios Mass Cytometer (Fluidigm, South San Francisco, CA, USA)
Software	Data normalization, concatenation, and debarcoding were performed using CyTOF software (Fluidigm Co.). Manual gating on raw data files was carried out using FlowJo v10 (FlowJo LLC., Ashland, OR).
Cell population abundance	Cells were plated in a round-bottom, 96-well plate at the density of 3e6 cells in culture with 300 mL RPMI, 5% FBS, Pen-Strep per well.
Gating strategy	Gating Strategy is available along with FCS files at FlowRepository (FR-FCM-Z4MA and FR-FCM-Z2V9). Wherein, experiment ID FR-FCM-Z4MA may be accessed via the following URL: https://flowrepository.org/id/RvFrOki98cB1R0srOsojhr4rTJpjCchGopou7h7BCE2CAS6fYgNWUdFLnbCSEO38. Please share this URL with the reviewers.

- [x] Tick this box to confirm that a figure exemplifying the gating strategy is provided in the Supplementary Information.
